# Mitochondria-associated membrane collapse impairs TBK1-mediated proteostatic stress response in ALS

**DOI:** 10.1073/pnas.2315347120

**Published:** 2023-11-15

**Authors:** Seiji Watanabe, Yuri Murata, Yasuyoshi Oka, Kotaro Oiwa, Mai Horiuchi, Yohei Iguchi, Okiru Komine, Akira Sobue, Masahisa Katsuno, Tomoo Ogi, Koji Yamanaka

**Affiliations:** ^a^Department of Neuroscience and Pathobiology, Research Institute of Environmental Medicine, Nagoya University, Nagoya, Japan; ^b^Department of Genetics, Research Institute of Environmental Medicine, Nagoya University, Nagoya, Japan; ^c^Department of Neurology, Nagoya University Graduate School of Medicine, Nagoya, Japan; ^d^Medical Interactive Research and Academia Industry Collaboration Center, Research Institute of Environmental Medicine, Nagoya University, Nagoya, Japan; ^e^Institute for Glyco-core Research, Nagoya University, Nagoya, Japan; ^f^Center for One Medicine Innovative Translational Research, Nagoya University, Nagoya, Japan

**Keywords:** mitochondria-associated membrane, sigma-1 receptor, TANK-binding kinase 1, stress granules, amyotrophic lateral sclerosis

## Abstract

The organelle contact site of the endoplasmic reticulum and mitochondria, known as the mitochondria-associated membrane (MAM), is a multifunctional microdomain in cellular homeostasis. We previously reported that MAM disruption is a common pathological feature in amyotrophic lateral sclerosis (ALS); however, the precise role of MAM in ALS was uncovered. Here, we show that the MAM is essential for TANK-binding kinase 1 (TBK1) activation under proteostatic stress conditions. A MAM-specific E3 ubiquitin ligase, autocrine motility factor receptor, ubiquitinated nascent proteins to activate TBK1 at the MAM, which results in ribosomal protein degradation. MAM or TBK1 deficiency under proteostatic stress conditions resulted in increased cellular vulnerability in vitro and motor impairment in vivo. Thus, MAM disruption exacerbates proteostatic stress via TBK1 inactivation in ALS. Our study has revealed a proteostatic mechanism mediated by the MAM–TBK1 axis, highlighting the physiological importance of the organelle contact sites.

## Significance

Recent studies, including ours, have suggested that mitochondria-associated membrane (MAM) disruption is a common pathogenic mechanism in amyotrophic lateral sclerosis (ALS). In the present study, we have revealed that TANK-binding kinase 1 (TBK1) activity is markedly reduced in the lesions of sporadic ALS patients and ALS model mice, which is associated with MAM disruption. We also found the molecular mechanistic link between the MAM and TBK1 activation under proteostatic stress conditions mediated by MAM-specific ubiquitination. These findings propose a critical role of the MAM in ALS pathogenesis. Moreover, our findings emphasize the importance of organelle contact sites in cellular homeostasis and will provide further insights into therapeutic options targeting the MAM integrity.

Organelle contact sites between the endoplasmic reticulum (ER) and mitochondria, known as the mitochondria-associated membranes (MAMs), are multifunctional microdomains in cellular homeostasis (1, 2). The MAMs are highly dynamic structures within the cell, and they increase or decrease in abundance in response to the environment (3). MAM dysregulation has been linked to various neurological diseases, including amyotrophic lateral sclerosis (ALS) (4, 5). However, it is not yet clear how MAM disruption leads to neurodegeneration.

ALS is a fatal neurodegenerative disease characterized by the selective loss of motor neurons (6). Most ALS patients are sporadic, i.e., they have no genetic background; however, in familial ALS patients, more than 20 genes have been identified as the causative genes of ALS. We previously reported that MAM collapse was a common pathology in *SOD1*- and *SIGMAR1*-linked ALS (7). Sigma-1 receptor (σ1R), a gene product of the *SIGMAR1* gene, is a MAM-specific chaperone protein, the deficiency of which is a cause of juvenile ALS (ALS16) (8). Mutations in the *SIGMAR1* gene also cause distal hereditary motor neuropathy (9), suggesting that σ1R function is closely associated with motor neuronal function. In *SOD1*- or *SIGMAR1*-linked ALS, MAM disruption leads to Ca^2+^ dysregulation, resulting in reduced ATP synthesis and Ca^2+^-dependent cell death (7). In previous research, we established a quantitative high-throughput reporter, MAMtracker-Luc, and showed that MAM disruption is widely induced by the causative genes of familial ALS (9). Although Ca^2+^ dysregulation is one possible mechanism of ALS pathogenesis, it is not yet clear whether other mechanisms are involved in this pathological process.

*TBK1*, which encodes TANK-binding kinase 1 (TBK1), has been identified as a causative gene in ALS with frontotemporal lobar degeneration (FTLD-ALS4) (10, 11). Nonsense or frameshift mutations in *TBK1* reduce the expression levels of TBK1 at the RNA and protein levels (12, 13), suggesting that TBK1 haploinsufficiency is the mechanism responsible for the *TBK1*-linked ALS. However, it has yet to be determined whether TBK1 loss-of-function is involved in the pathomechanism of ALS without TBK1 mutations, such as in sporadic ALS patients.

TBK1 is best known as a regulator in innate immunity (14). Stimuli that activate innate immunity, such as cytosolic double-strand DNA or lipopolysaccharides, activate TBK1 following recognition by stimulator of interferon genes (STING) or Toll-like receptors, respectively. Activated TBK1 phosphorylates interferon regulatory factor 3 (IRF3), a transcription factor that induces inflammatory cytokines, thereby promoting dimerization and nuclear translocation of IRF3, which in turn increases the expression of various inflammatory factors. In addition, TBK1 regulates autophagy (15, 16). TBK1 phosphorylates SQSTM1/p62, an adaptor protein for autophagic protein degradation, and promotes the binding of p62 to its degradation targets, i.e., polyubiquitinated proteins (17). This function helps rapidly eliminate foreign bacteria or exogenous viral components by autophagy, enhancing the cellular defense against infection (18). TBK1-dependent autophagy is also involved in the clearance of misfolded proteins in ALS (19).

In ALS, the mitochondria are one of the primary targets for injury in motor neurons (6). ALS induces mitochondrial dysfunction represented by compromised mitochondrial energy synthesis, increased production of reactive oxygen species, and altered mitochondrial dynamics. MAM alternation is also involved in mitochondrial fragmentation in ALS associated with the Sig1R loss-of-function (20). TBK1 contributes to the elimination of such damaged mitochondria via selective autophagy, called mitophagy, by phosphorylating optineurin (21) or Rab7A (22). Moreover, TBK1 loss-of-function is associated with impaired mitophagy in ALS patients (23). These findings suggest the important role of TBK1 in the maintenance of the mitochondria, which is disrupted in neurodegenerative diseases.

Despite these findings, the physiological role of TBK1, especially in the central nervous system (CNS), remains largely unknown. In the present study, we aimed to elucidate the role of TBK1 at the MAM. We found that TBK1 activity is dependent on MAM integrity and is induced by MAM-specific accumulation of polyubiquitinated proteins under proteotoxic conditions. Moreover, disruption of this pathway induced cellular vulnerability to proteotoxicity. Our study shows that TBK1 regulates the cellular proteostatic response at the MAM, highlighting the physiological importance of organelle contact sites.

## Results

### TBK1 Is Inactive in Human Sporadic ALS Patients and ALS Model Mice.

**Fig. 1.**
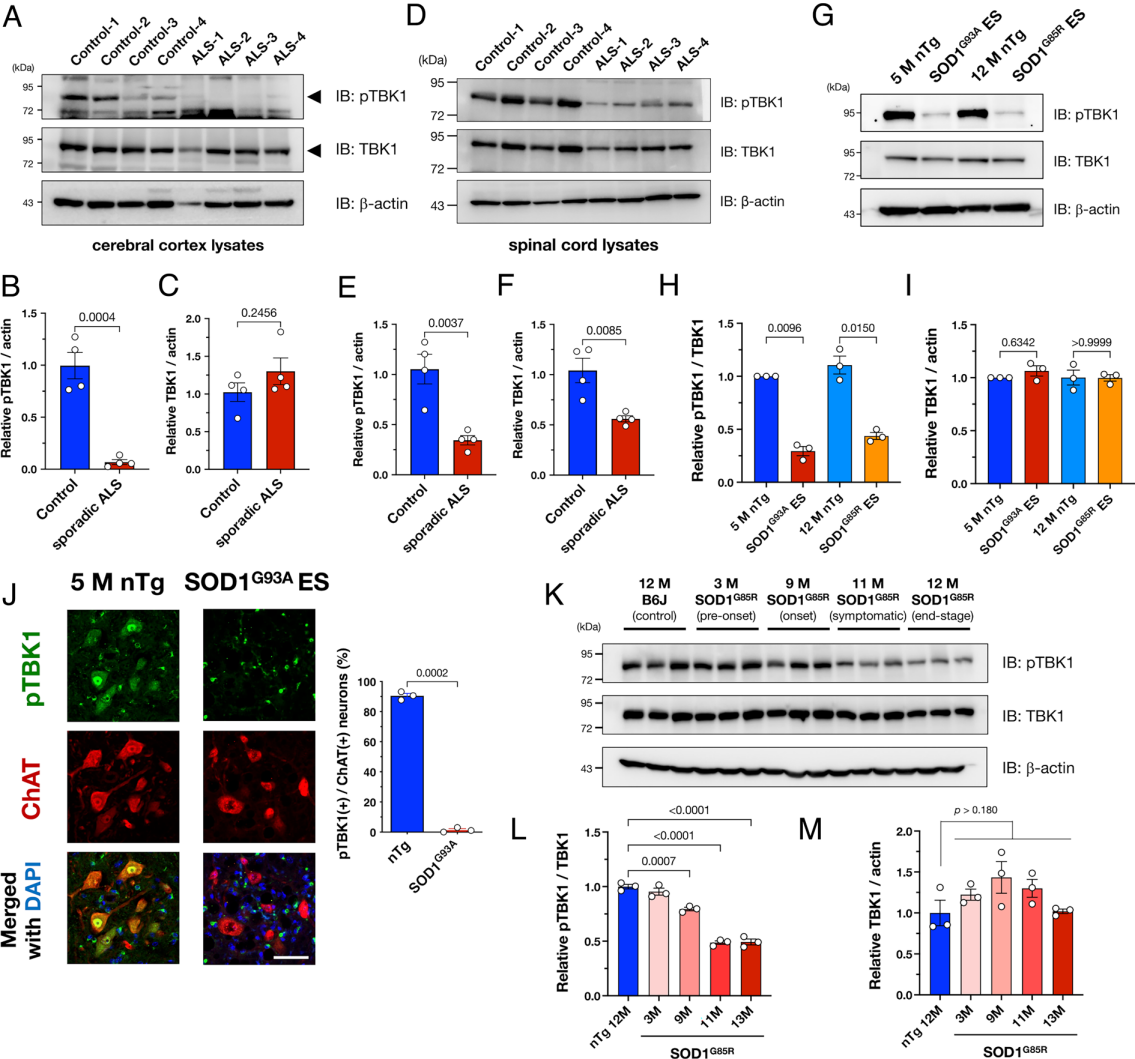
TANK-binding kinase 1 (TBK1) is inactive in the brains and spinal cords of sporadic ALS patients or SOD1-linked ALS model mice. (*A*–*F*) TBK1 was inactive in the cerebral cortices and the spinal cords of sporadic ALS patients. Representative immunoblotting images of brain (*A*–*C*) or spinal cords (*D*–*F*) lysates from four sporadic ALS patients and control patients. Levels of active TBK1 (TBK1 phosphorylated at Ser172; pTBK1) were significantly reduced in ALS patients (*B* and *E*). Total TBK1 protein levels were reduced in the ALS spinal cords (*F*) but unaffected in the brains (*C*). (*G*–*J*) TBK1 was inactive in the spinal cords of SOD1-linked ALS model mice. Representative immunoblotting images of spinal cord lysates from two different SOD1-linked ALS model mice, carrying p.Gly93Ala (SOD1^G93A^) or p.Gly85Arg (SOD1^G85R^), at the end stage (*G*). Similar to the ALS patients’ tissues, pTBK1 was specifically decreased in ALS model mice (*H*) without degradation of the TBK1 protein itself (*I*). pTBK1 was predominantly downregulated in the motor neurons of SOD1^G93A^ mice, which are indicated by choline acetyltransferase (ChAT), at the end stage (*J*) (Scale bar, 50 µm). (*K*–*M*) TBK1 inactivation was associated with disease onset in the SOD1^G85R^ ALS model mouse brain. pTBK1 levels began to decrease from the onset of the disease (9 M) (*K* and *L*) without a reduction in the total TBK1 protein level (*M*). Relative levels of pTBK1 (*B*, *E*, *H*, and *L*) and TBK1 (*C*, *F*, *I*, and *M*) were quantified from the corresponding immunoblots and are plotted as means ± SEM with *P*-values.

Although TBK1 haploinsufficiency is linked to inherited forms of ALS with a mutation in the *TBK1* (13), it was not yet clear whether TBK1 dysregulation was apparent in sporadic ALS patients, which account for >90 % of ALS cases. Thus, to achieve clarification in sporadic ALS cases, we performed immunoblotting using the postmortem tissues of sporadic ALS patients and found that levels of an active form of TBK1, which is phosphorylated at Ser 172 (pTBK1), were significantly decreased in the brains (Fig. 1 *A*–*C*) and spinal cords (Fig. 1 *D*–*F*) of the patients with ALS. TBK1 was also inactivated in the spinal cords of *SOD1*-linked ALS model mice at the end stage (Fig. 1 *G*–*I*). Immunofluorescence results showed that pTBK1 was mainly localized in the ventral horn motor neurons and that pTBK1 levels were specifically reduced in end-stage SOD1^G93A^ mice (Fig. 1*J*). In addition, endogenous TBK1 was highly activated in the CNS (*SI Appendix*, Fig. S1). In the wild-type mice, although pTBK1 was widely observed in neurons, including nonmotor neurons, the pTBK1 level was highest in motor neurons (*SI Appendix*, Fig. S2). Thus, the reduction in pTBK1 levels appeared to be associated with neurodegeneration in ALS. Consistent with this notion, pTBK1 levels began to decrease at the onset of disease in SOD1^G85R^ ALS model mice (Fig. 1 *K*–*M*). The pTBK1 levels were not altered between young (5 mo old) and old (12 mo old) mouse spinal cords (*SI Appendix*, Fig. S3), suggesting that normal aging does not affect pTBK1 levels in the CNS. In addition, pTBK1 puncta in non-neuronal cells seemed to be a non-specific immunoreactivity in oligodendrocytes (*SI Appendix*, Fig. S4). Although the total amount of TBK1 protein was slightly reduced in the ALS spinal cords (Fig. 1*F*), the TBK1 protein levels remained unaltered in the brains of the patients (Fig. 1*C*) and ALS mouse models (Fig. 1*I*), suggesting that inactivation of TBK1 rather than degradation of TBK1 protein predominantly caused the reduction in pTBK1 levels. Overall, these results suggest that TBK1 insufficiency is a common pathological feature of ALS.

### σ1R Is Essential for TBK1 Activation.

**Fig. 2.**
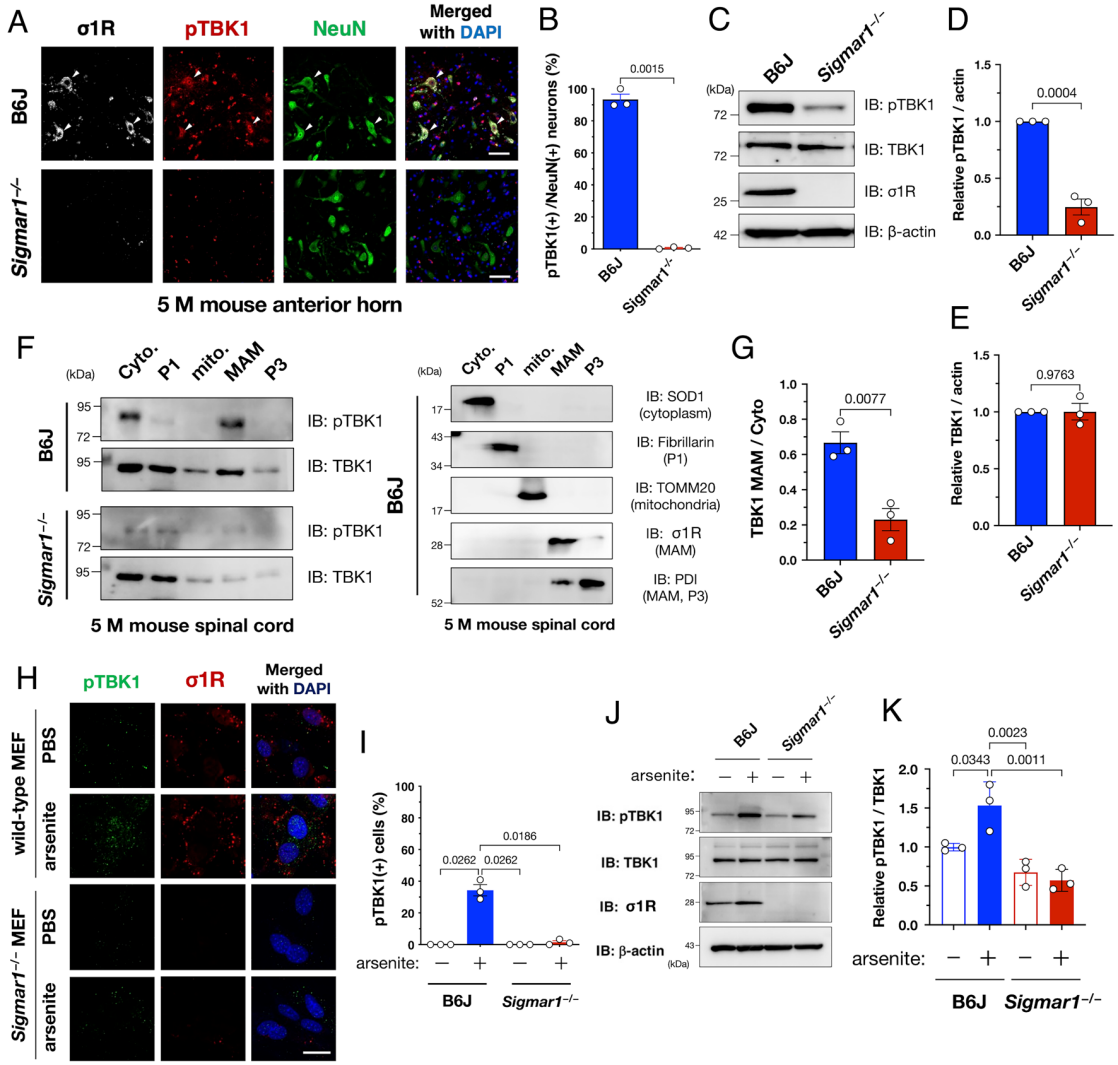
Sigma-1 receptor (σ1R) is essential for TBK1 activation in mouse spinal cords or cells under arsenite-induced stress. (*A* and *B*) TBK1 was inactive in the lumbar ventral horn neurons of *Sigmar1* (which encodes murine σ1R), knockout (*Sigmar1^−/−^*) mice. Arrowheads in (*A*) indicate TBK1-active neurons in a wild-type mouse. Percentage of pTBK1-positive neurons in (*A*) is plotted (*B*). (*C*–*E*) pTBK1 levels were reduced without degradation in the *Sigmar1^−/−^* mouse spinal cords. Representative immunoblotting images are shown in (*C*). Relative levels of pTBK1 and TBK1 were quantified in (*D*) and (*E*), respectively. (*F* and *G*) pTBK1 was localized at the mitochondria-associated membranes (MAMs), where σ1R is specifically localized. Cyto., cytoplasm; P1, nuclei and debris; mito., mitochondria; MAM; and P3, microsomes. Representative immunoblotting images for pTBK1, TBK1, and the specific markers used to validate appropriate fractionation (*F*). Relative levels of TBK1 in the MAM fraction normalized to the one in the cytoplasmic fraction are shown in (*G*). (*H*–*K*) Activation of TBK1 by arsenite treatment (0.1 mM, 30 min) requires σ1R in mouse embryonic fibroblasts (MEFs) from wild-type or *Sigmar1^−/−^* mice. Representative immunocytochemical (*H*) and immunoblotting (*J*) images are shown with quantification of the relative pTBK1-positive cell numbers or protein levels in (*I*) and (*K*), respectively. The data are expressed as means ± SEM; *P*-values are shown. Scale bars, 50 µm in (*A*), 20 µm in (*H*).

We previously found that σ1R dysfunction-associated MAM disruption was involved in the disease onset of ALS (7); thus, we examined the potential association between σ1R-associated MAM disruption and TBK1 inactivation in ALS. We found that pTBK1 levels were substantially decreased in σ1R knockout (*Sigmar1^−/−^*) mouse spinal cord neurons (Fig. 2 *A* and *B*), indicating that σ1R is required to maintain TBK1 activity. TBK1 inactivation in *Sigmar1^−/−^* mice, i.e., a lack of TBK1 degradation, was confirmed using immunoblotting analyses (Fig. 2 *C*–*E*). Moreover, pTBK1 specifically accumulated at the MAM in the presence of σ1R (Fig. 2 *F* and *G*). Therefore, σ1R-associated MAM is apparently essential to maintaining the TBK1 activity in the spinal cord neurons.

In contrast to the high activation of TBK1 in the spinal cord neurons, endogenous TBK1 was generally inactivated in cultured cell lines, including human cervical carcinoma HeLa, mouse neuroblastoma Neuro2a, and human neuroblastoma SH-SY5Y (*SI Appendix*, Fig. S5). Neurotrophic factors also failed to activate TBK1 in a σ1R-dependent manner (*SI Appendix*, Fig. S6). However, treatment with sodium arsenite, an oxidative stress inducer, activated TBK1 in a σ1R-dependent manner in primary mouse embryonic fibroblasts (MEFs) from the wild-type or *Sigmar1^−/−^* mice (Fig. 2 *H*–*K*). Similar observation was confirmed in HeLa cells (*SI Appendix*, Fig. S7). Thus, sodium arsenite treatment successfully mimicked σ1R-dependent TBK1 activation in cultured cells.

### Proteostatic Stress Induces TBK1 Translocation and Activation at the MAM.

**Fig. 3.**
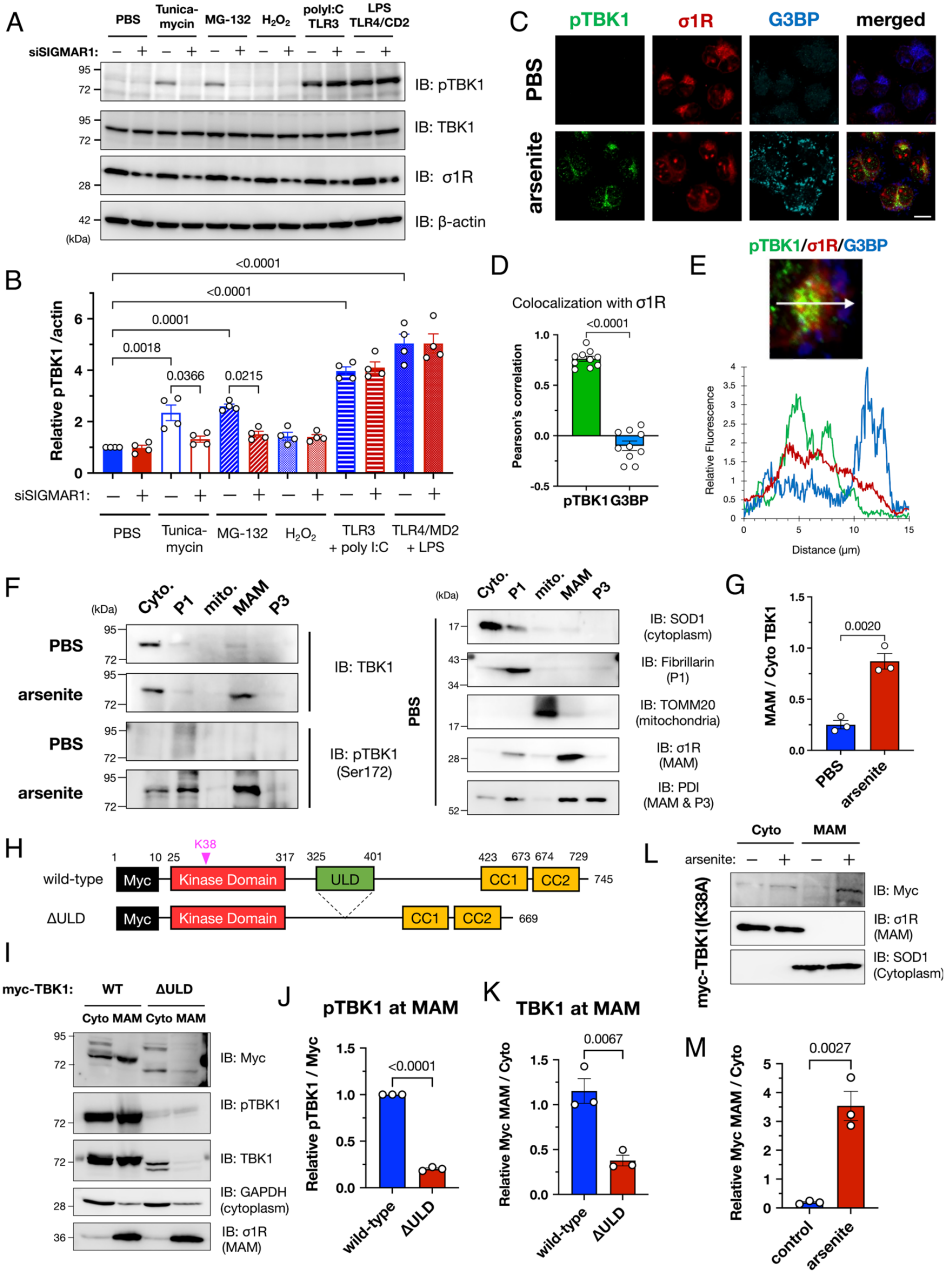
TBK1 was activated at the MAM under proteostatic stress conditions, including arsenite treatment. (*A* and *B*) σ1R was crucial for TBK1 activation under proteostatic stress conditions. HeLa cells with or without siSIGMAR1 were treated with tunicamycin (5 µg/mL; ER stress), MG-132 (1 µM; proteasomal inhibition), hydrogen peroxide (100 µM; H_2_O_2_; oxidative stress), poly I:C [100 µM; Toll-like receptor 3 (TLR3) agonist], or lipopolysaccharide [1 µg/mL; LPS; Toll-like receptor 4 (TLR4) agonist] for 6 h. Human TLR3 or TLR4 with the coactivator MD2 were overexpressed one day before the treatment with agonists. Representative immunoblotting images (*A*) and relative levels of pTBK1 (*B*) are provided. (*C*–*E*) pTBK1 colocalized with σ1R. Representative immunofluorescent images are shown in (*C*). HeLa cells were treated with 0.1 mM arsenite for 30 min. Colocalization coefficiency (*D*) and fluorescent intensity profile (*E*) are also provided; whereas pTBK1 was colocalized with σ1R, suggesting pTBK1 localization at the MAM, Ras GTPase–activating protein-binding protein 1 (G3BP), a marker of stress granules (SGs), was close but independent of the MAM (Scale bar, 10 µm). (*F* and *G*) TBK1 translocated into the MAM by arsenite treatment (0.1 mM, 30 min). Representative immunoblotting images for TBK1, pTBK1, and the specific markers used to validate appropriate fractionation (*F*) with quantification of the relative TBK1 levels at the MAM normalized to the one in the cytoplasm (*G*). (*H*) Schematic diagram of TBK1 and its mutant lacking the ubiquitin-like domain (ΔULD). Known functional domains are indicated by colored rectangles. Myc, c-Myc tag; CC1/2, coiled-coil domain 1/2. (*I*–*K*) ULD of TBK1 was essential for translocation into the MAM. Representative immunoblotting images (*I*) are shown with quantification of the relative pTBK1 (*J*) and total TBK1 (*K*) levels at the MAM. (*L* and *M*) Phosphorylation of TBK1 is not essential for TBK1 translocation into the MAM. TBK1 carrying the p.K38A mutation, a kinase-dead mutant, was translocated into the MAM after the arsenite treatment. Representative immunoblotting images (*L*) are shown with quantification of the relative TBK1 levels at the MAM normalized to the one in the cytoplasm (*M*). All the data are expressed as means ± SEM with *P*-values.

To identify other stimuli that induce TBK1 activation in vitro, we treated HeLa cells with various stressors (Fig. 3 *A* and *B*). Two proteostatic stress inducers, tunicamycin (ER stress inducer) and MG-132 (proteasomal inhibitor), significantly activated TBK1. In contrast, hydrogen peroxide–induced oxidative stress did not activate TBK1. In a previous study, arsenite treatment induced not only oxidative stress but also affected protein folding to induce proteostatic stress (24). Taken together, these results indicate that TBK1 is activated by proteostatic stress, including that caused by arsenite treatment. Interestingly, Toll-like receptor (TLR) 4–mediated innate immune responses led to increased activation of TBK1, whereas TLR-mediated TBK1 activation was unaffected by σ1R depletion. In addition, siRNA-mediated suppression of TLR3, TLR4, or STING, another major activator of TBK1, did not affect TBK1 activation by arsenite treatment (*SI Appendix*, Fig. S8). These findings indicate that innate immune pathways are independent of MAM/σ1R-dependent TBK1 activation.

Under proteostatic stress conditions, TBK1 was translocated into the MAM (Fig. 3 *C*–*G*). Immunocytochemical analyses revealed that pTBK1 colocalized with σ1R (Fig. 3 *C* and *D*). Arsenite is known to prevent cellular translation and promote the formation of small membraneless cytosolic compartments composed of RNA and ribonuclear proteins known as stress granules (SGs) (25). Although Ras GTPase–activating protein-binding protein 1 (G3BP), an SG marker, was not colocalized with σ1R or pTBK1, they were located in close proximity (Fig. 3*E*). TBK1 contains four functional domains: a kinase domain, a ubiquitin-like domain (ULD), and two coiled-coil domains (CC1 and CC2) (Fig. 3*H*). In particular, the ULD is required for interactions with ubiquitin and ubiquitin-associated adaptor proteins such as p62 (26, 27). Moreover, local accumulation of TBK1 through binding to ubiquitinated proteins would cause TBK1 activation by a transautophosphorylation mechanism (26, 28). Thus, to investigate whether the ULD domain was involved in MAM translocation of TBK1, we prepared a ULD-lacking TBK1 mutant (ΔULD). Although the ΔULD mutant was extremely unstable, we confirmed that the ΔULD mutant was not localized at the MAM and predominantly localized in the cytoplasm (Fig. 3 *I*–*K*). Consistent with this observation, even K38A TBK1 mutants, a kinase-deficient mutant, translocated into the MAM (Fig. 3 *L* and *M*), suggesting that the TBK1 recruitment into the MAM is independent of its activation. These data indicate that TBK1 is translocated into the MAM under proteostatic stress conditions, possibly by binding to polyubiquitinated proteins accumulated at the MAM.

### Autocrine Motility Factor Receptor/gp78-mediated MAM-specific Ubiquitination Is Crucial for TBK1 Activation under Proteostatic Stress Conditions.

**Fig. 4.**
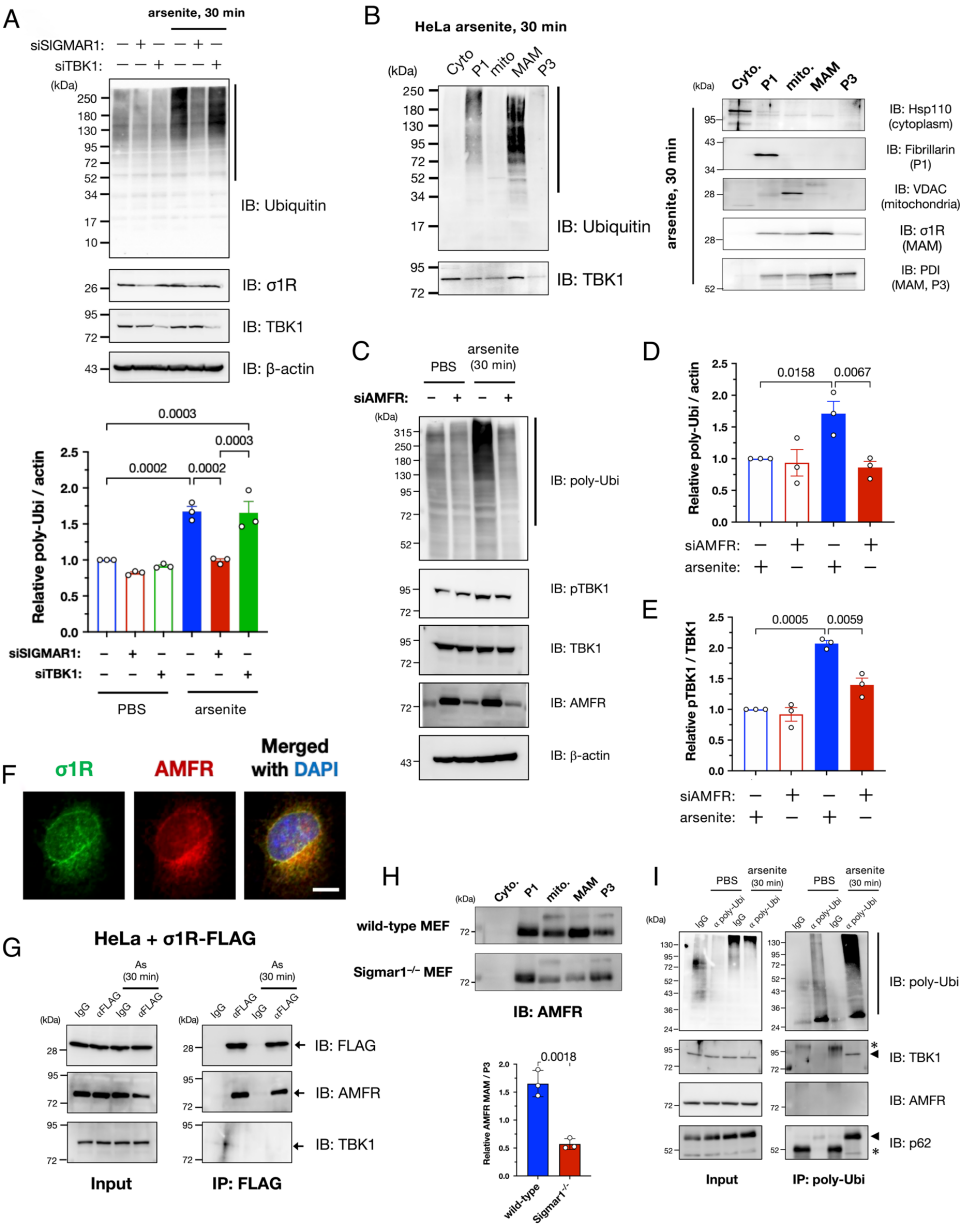
Autocrine motility factor receptor (AMFR)/gp78 is essential for MAM-specific ubiquitination induced by arsenite. (*A*) Arsenite-induced accumulation of polyubiquitinated proteins (pUbi) was σ1R-dependent. HeLa cells were transfected with siSIGMAR1 and/or siRNA against *TBK1* (siTBK1) 1 d before arsenite treatment (As: 0.1 mM for 30 min). Representative immunoblotting images (*Upper*) and quantification of pUbi (lower; quantified the band intensities indicated by the black line in the upper image). (*B*) Arsenite-induced pUbi were predominantly accumulated at the MAM after arsenite treatment (0.1 mM for 30 min). Specific markers were used to confirm appropriate fractionation (*Right*). (*C*–*E*) AMFR was crucial for arsenite-induced ubiquitination. HeLa cells were transfected with siRNAs against *AMFR* (siAMFR) 1 d before arsenite treatment (As: 0.1 mM for 30 min). Representative immunoblotting images (*C*) with quantification of relative pUbi (*D*) and pTBK1 (*E*) levels. pTBK1 levels were also reduced by siAMFR. (*F*–*H*) AMFR interacted with σ1R to localize at the MAM. Representative immunofluorescent images of σ1R and AMFR in HeLa cells (*F*). Coimmunoprecipitation was performed using C-terminally FLAG-tagged σ1R (σ1R-FLAG); AMFR and σ1R interacted, and the interaction was not affected by arsenite (*G*). AMFR was lost in the MAM fraction of *Sigmar1^−/−^* MEFs (*H*), suggesting that σ1R functions as a scaffold for AMFR. Relative AMFR levels at the MAM [*Lower* panel in (*H*)] (Scale bar, 10 µm). (*I*) pUbi induced by arsenite interacted with TBK1 and SQSTM1/p62. HeLa cell lysates were coimmunoprecipitated using an antibody against pUbi. Representative immunoblots show bound TBK1 and SQSTM1/p62 proteins in arsenite-treated HeLa cells. Asterisks indicate nonspecific bands. All the data are expressed as means ± SEM with *P*-values.

We also investigated how the MAM contributes to TBK1 activation under proteostatic stress conditions. As shown in Fig. 4*A*, σ1R knockdown prevented the accumulation of ubiquitinated proteins caused by arsenite treatment. However, TBK1 suppression did not prevent the arsenite-induced ubiquitination, implying that ubiquitination at the MAM precedes TBK1 activation. Notably, ubiquitinated proteins accumulated predominantly at the MAM (Fig. 4*B*). Given that the ULD domain, which is essential for TBK1 activation (Fig. 3 *F*–*I*), is a ubiquitin-binding motif, this collective evidence suggests that accumulation of MAM-specific ubiquitinated proteins recruits TBK1 to the MAM and promotes its activation by increasing the local TBK1 concentration.

However, the question remained as to how MAM-specific ubiquitination occurred. Hence, we studied ER-mediated degradation of stress-induced misfolded proteins via the ubiquitin-proteasome system, i.e., ER-associated degradation (ERAD) (29), because some ERAD-associated proteins are accumulated at the MAM (1, 30). Among the E3 ubiquitin ligases involved in ERAD, we found that autocrine motility factor receptor (AMFR)/gp78 depletion predominantly reduced the accumulation of the polyubiquitinated proteins following arsenite treatment (Fig. 4 *C* and *D* and *SI Appendix*, Fig. S9). When AMFR was depleted, TBK1 activation was inhibited along with the reduction in polyubiquitinated proteins (Fig. 4 *C* and *E*). Thus, AMFR was seemingly responsible for MAM-specific ubiquitination. Additionally, we confirmed that AMFR colocalized with σ1R (Fig. 4*F*), interacted with σ1R independent of arsenite treatment (Fig. 4*G*), and was significantly reduced in terms of amounts of AMFR in the MAM of *Sigmar1^−/−^* MEFs (Fig. 4*H*). Collectively, these data suggest that σ1R anchors AMFR in the MAM. Furthermore, TBK1 and p62 were coimmunoprecipitated with polyubiquitinated proteins but not with AMFR (Fig. 4*I*), providing evidence for an indirect mechanism of TBK1 recruitment into the MAM by AMFR-mediated ubiquitination rather than through a direct interaction of TBK1 with the ERAD complexes at the MAM.

### AFMR and TBK1 Target Proteins under Synthesis, including Ribosomal Complexes.

**Fig. 5.**
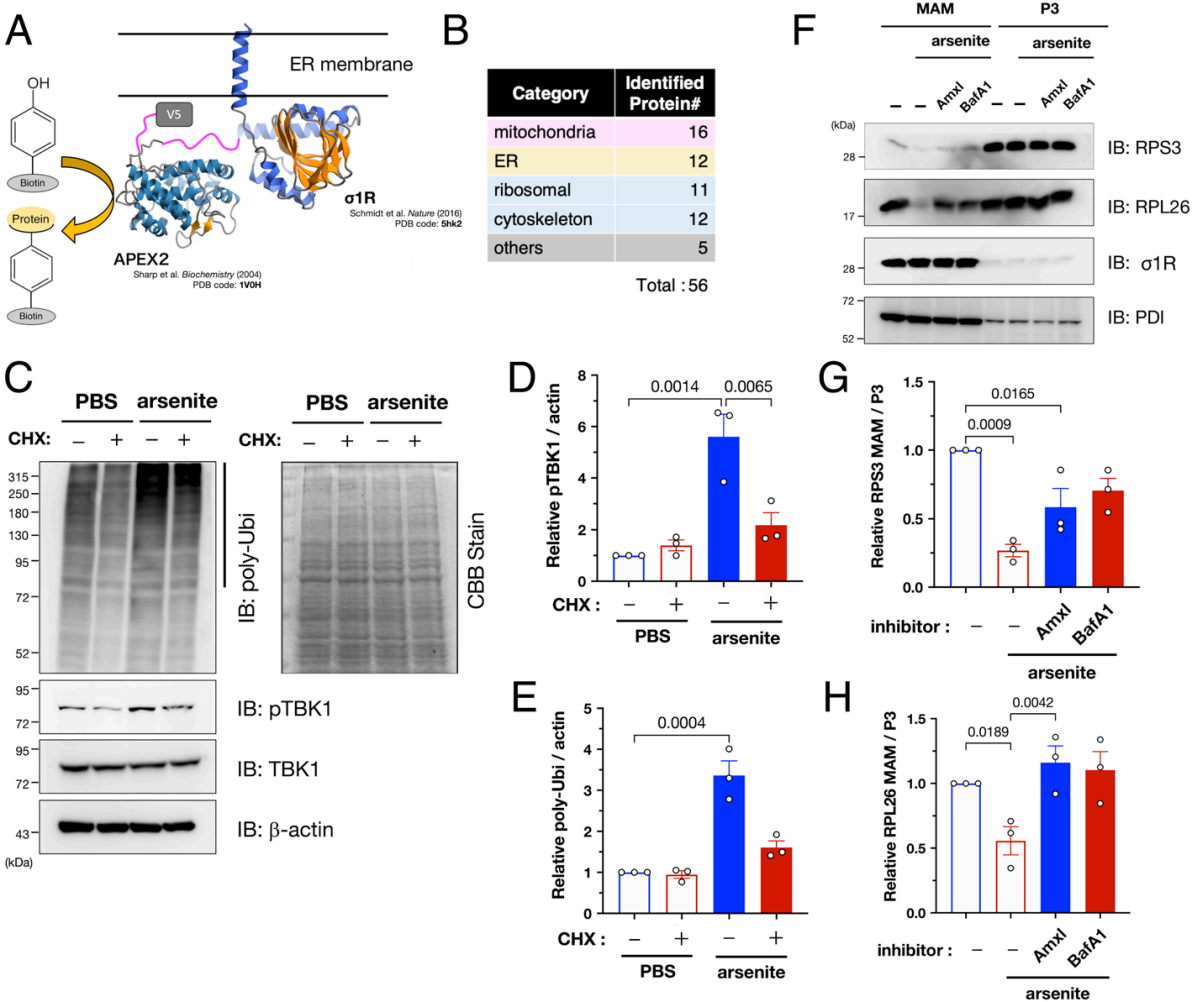
AMFR targets nascent proteins and TBK1 induces degradation of ribosomal proteins at the MAM under arsenite-induced stress conditions. (*A*) Schematic illustration of the APEX2-based proximal labeling assay including σ1R C-terminally fused to APEX2-V5 (σ1R-APEX2) (*Upper*). σ1R and APEX structure were from PDB 5hk2 and 1V0H, respectively. (*B*) Identified proteins were classified as shown in the table (the full list of the identified proteins is available in Dataset S1). (*C*–*E*) AMFR targets nascent proteins. HeLa cells were treated with cycloheximide (50 µg/mL; CHX) for 1 h before arsenite treatment (0.1 mM for 30 min), resulting in reduced pUbi and pTBK1. Representative immunoblotting images (*C*, *Left* panel). No effect of CHX on total loading amounts was confirmed by Coomassie brilliant blue staining (*C*, *Right* panel). Relative pTBK1 (*D*) and pUbi (*E*) levels were quantified. (*F*–*H*) Ribosomal proteins were specifically degraded by TBK1-dependent autophagy at the MAM. HeLa cells were pretreated for 1 h with amlexanox (3.3 µM; Amlx), a TBK1 inhibitor, or bafilomycin A1 (100 nM; BafA1), an autophagy inhibitor, before arsenite treatment (0.1 mM, 30 min) MAM and P3 fractions were analyzed by immunoblotting using indicated antibodies (*F*). The levels of two ribosomal subunits, RPS3 (*G*) and RPL26 (*H*), at the MAM were quantified in three independent experiments. All the data are expressed as means ± SEM with *P*-values.

To identify the target proteins of MAM-specific ubiquitination, we performed a proximal labeling assay using engineered ascorbate peroxidase APEX2 fused to the C-terminus of σ1R (σ1R-APEX2; Fig. 5*A*), similar to recent studies (31, 32). σ1R-APEX2 specifically labeled proteins closely associated with σ1R (*SI Appendix*, Fig. S10 *A* and *B*). The 56 labeled proteins (listed in Dataset S1) were mainly related to the mitochondria [16/56 (29 %)] and ER [12/56 (21 %)], indicating that MAM proteins were successfully labeled in the assay (Fig. 5*B*). Intriguingly, the identified proteins also contained various ribosomal subunits, suggesting that AFMR may ubiquitinate the nascent proteins at the MAM. To test this hypothesis, we treated the cells with cycloheximide (CHX) to prevent translation before arsenite treatment; thus, we confirmed that CHX significantly reduced ubiquitination and TBK1 activation (Fig. 5 *C*–*E*). Arsenite reportedly induces ribophagy, i.e., ribosomal degradation via selective autophagy (33). Therefore, we speculated that the observed ubiquitination was associated with autophagy-dependent degradation of ribosomes at the MAM. Consistent with this notion, levels of the ribosomal subunits RPS3 and RPL26 were reduced at the MAM following arsenite treatment, but this reduction was prevented by amlexanox, a TBK1-specific inhibitor, or bafilomycin A1, an autophagy inhibitor (Fig. 5 *F*–*H*). In addition, RPS3 and RPL26 reduction at the MAM was also inhibited in AMFR- or TBK1-depleted cells (*SI Appendix*, Fig. S10*C*). The levels of RPS3 or RPL26 in non-MAM fractions were unchanged (*SI Appendix*, Fig. S11), supporting the notion that these ribosomal subunits are degraded at the MAM. Therefore, it is likely that ribosomal subunits, at least RPS3 and RPL26, are degraded in a TBK1-related autophagy-dependent manner. Notably, TBK1-dependent degradation of RPS3 and RPL26 was MAM-specific, and microsomal fractions, i.e., P3 fractions, were not entirely affected. Thus, AMFR apparently ubiquitinates proteins under synthesis at the MAM, TBK1 is recruited through the accumulation of polyubiquitinated proteins, and activated TBK1 induces autophagy, which contributes to ribosomal degradation.

### σ1R or TBK1 Deficiency Delays SG Formation In Vitro.

**Fig. 6.**
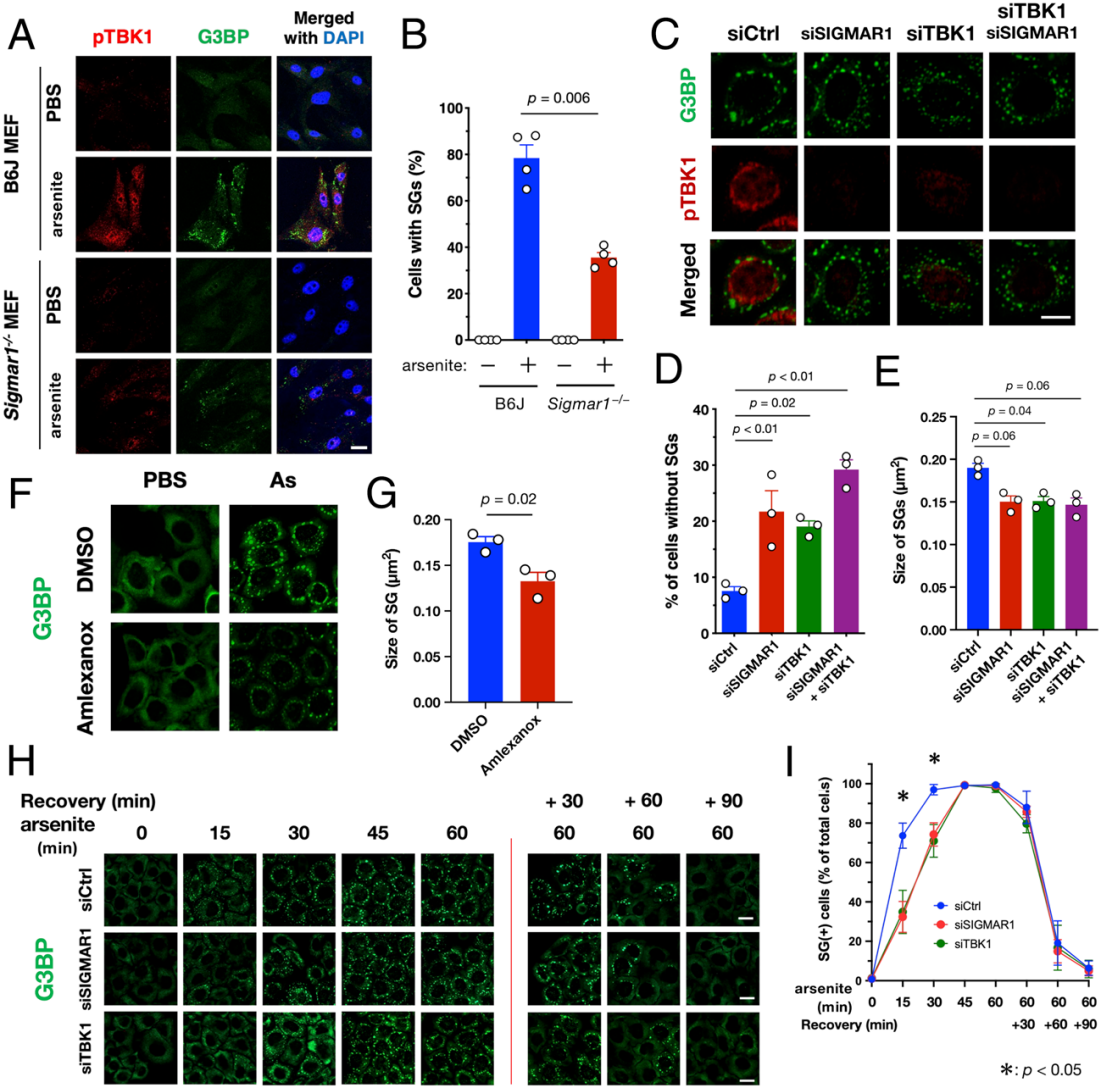
TBK1 activation at the MAM promotes SG formation. (*A* and *B*) σ1R-deficiency prevented SG formation. Wild-type or *Sigmar1^−/−^* MEFs were treated with arsenite (As: 0.1 mM for 30 min). Representative immunofluorescent images of HeLa cells stained with the antibodies for pTBK1 and G3BP (*A*). The percentage of cells with G3BP-positive granules was calculated and plotted as “Cells with SGs” (*B*). (*C*–*E*) Depletion of σ1R and/or TBK1 resulted in impaired SG formation. HeLa cells were treated with arsenite (0.1 mM for 30 min) with or without siSIGMAR1 and/or siTBK1. Representative immunofluorescent images of HeLa cells stained with the antibodies for pTBK1, G3BP (*C*). G3BP and TIAR were used as SG markers. The percentage of Cells with SGs was calculated and plotted (*D*). The size of the G3BP-positive SGs was also quantified (*E*). (*F* and *G*) Pharmacological inhibition of TBK1 using amlexanox [3.3 µM for 30 min before arsenite treatment (As: 0.1 mM for 30 min)] impaired SG formation in Hela cells. Representative immunofluorescent images (*F*) with quantification of SGs size (*G*). (*H* and *I*) σ1R or TBK1 deficiency delayed SG formation. HeLa cells were treated with arsenite (As: 0.1 mM) for the indicated times. After 60 min of incubation with arsenite, the cells were further incubated in growth medium (Recovery) for the indicated duration. Representative immunofluorescent images (*H*). The percentage of Cells with SGs was calculated and plotted as (*I*). All data are expressed as means ± SEM with *P*-values, and all the scale bars indicate 10 µm.

Because pTBK1 was located close to SGs (Fig. 3 *C*–*E*) according to G3BP marking, we used arsenite treatment to examine the effect of TBK1 defects in SG formation. We found that SG formation was impaired in *Sigmar1^−/−^* MEFs concomitant with TBK1 inactivation (Fig. 6 *A* and *B*). Additionally, siRNA-mediated depletion of σ1R or TBK1 reduced the number and average size of SGs in HeLa cells (Fig. 6 *C*–*E*). Pharmacological inhibition of TBK1 using amlexanox also reduced the size of SGs (Fig. 6 *F* and *G*), suggesting that TBK1 activity was responsible for normal SG formation. According to time course analyses, the TBK1 defect specifically delayed SG formation but did not affect SG disassembly (Fig. 6 *H* and *I*). A direct involvement of TBK1 in SG formation was also confirmed using TBK1-deficient MEFs (*SI Appendix*, Fig. S12). Given that autophagic degradation of ribosomal complexes is required for SG assembly (34), our findings suggest that impaired TBK1-dependent autophagy delays SG formation. Moreover, σ1R or TBK1 deficiency also compromised the stress granules’ formation induced by paraquat in mouse neuroblastoma Neuro2a cells (*SI Appendix*, Fig. S13). Thus, the TBK1-mediated proteostatic stress response does not appear to be specific to the particular cell type.

Since arsenite damages cells not only by proteostatic stress but also by multiple mechanisms, we asked whether the proposed pathways are general under proteostatic stress conditions. As shown in Fig. 3 *A* and *B*, we have demonstrated that tunicamycin, an ER stress inducer, causes TBK1 activation in a MAM-dependent manner. Hence, we intended to confirm our observations using tunicamycin. When we treated the cells with tunicamycin, 1) polyubiquitinated proteins accumulated at the MAM, 2) TBK1 translocated into the MAM and was activated in situ, and 3) σ1R suppression impaired SG formation in the cytoplasm (*SI Appendix*, Fig. S14). All these observations are well consistent with those of arsenite treatment, suggesting that the MAM–TBK1 axis is a general cellular response pathway against proteostatic stress.

### Impaired MAM-dependent TBK1 Activation Increases Cell Vulnerability In Vitro and Induces Motor Dysfunction In Vivo.

**Fig. 7.**
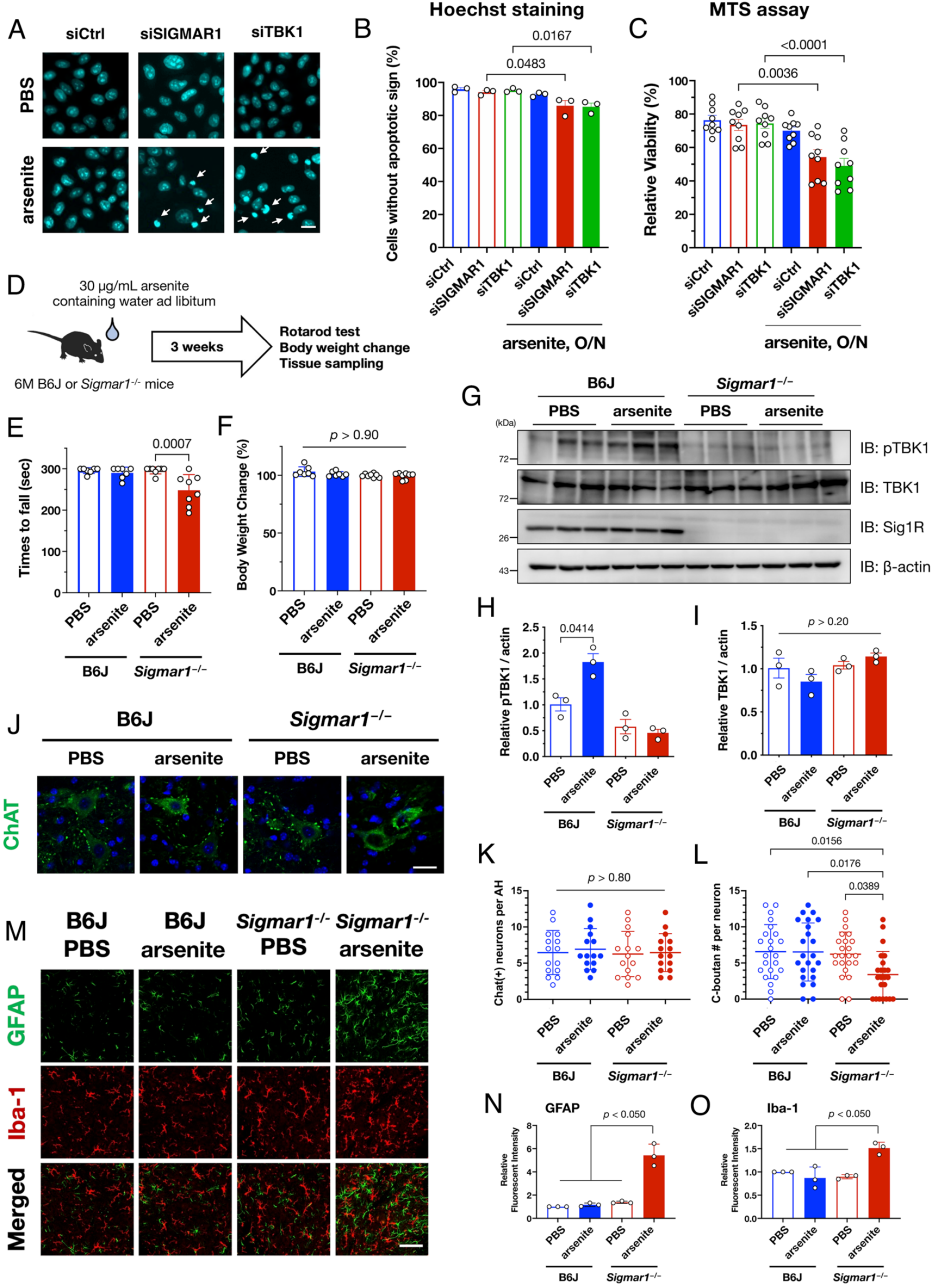
TBK1 inactivation is associated with stress vulnerability in vivo. (*A*–*C*) Impaired SG formation via loss of σ1R or TBK1 increased cell death. HeLa cells transfected with siSIGMAR1 or siTBK1 were treated with 0.1 mM arsenite overnight (O/N). Apoptotic cells, indicated by arrows, were visualized using Hoechst 33342 (*A*; Scale bar, 5 µm). The percentages of cells without an apoptotic phenotype are quantified and plotted (*B*). Relative cell viability evaluated by MTS assay is plotted (*C*). (*D*) Schematic diagram of arsenite oral administration. Water intake was around 6 mL/day; thus, mice ingested arsenite at approximately 10 mg/kg/day. (*E* and *F*) Rotarod score (*E*, a mean time staying on the rotating rod) and relative change in body weights (*F*) of arsenite-treated wild-type and *Sigmar1^−/−^* mice were plotted (*n* = 6 for B6J, 7 for *Sigmar1^−/−^*, each). Body weights relative to those at the starting point did not change, suggesting that the motor dysfunction was not due to acute systemic toxicity of arsenite. (*G*–*I*) Oral administration of arsenite increased pTBK1 levels in the brains of B6J but not *Sigmar1^−/−^* mice. Representative immunoblots (*G*) with quantification of relative pTBK1 (*H*) or total TBK1 (*I*) levels. (*J*–*L*) Arsenite treatment reduced the number of C-bouton without neurodegeneration. Immunohistochemistry using anti-ChAT antibody was performed to visualize motor neurons and C-boutons (*J*). The number of motor neurons (ChAT-positive cells) (*K*) and C-boutons per motor neuron (*L*) were quantified. A total of 15 ventral horns (*K*) or 23 motor neurons (*L*) from three independent mice were used for quantification. (*M*–*O*) Arsenite treatment induced astrogliosis in the ventral horn of arsenite-treated *Sigmar1^−/−^* mice. Relative signal intensities of astrocytes (GFAP) and microglia (Iba-1) were quantified per anterior horns of the mouse spinal cords in (*N*) and (*O*), respectively (*n* = 3) (Scale bar, 50 µm). The data in (*B*, *C*, *H*, and *I*) are expressed as means ± SEM, whereas other quantitative data are expressed as means ± SD; *P*-values are shown.

Because SGs are essential to the preservation of cellular tolerance under various stress conditions (35), we speculated that TBK1 defects following delays in SG formation might affect cellular vulnerability. Consistent with this notion, the number of apoptotic cells increased significantly when σ1R or TBK1 was knocked down using specific siRNAs (Fig. 7 *A* and *B*). Moreover, the cell viability measured using an MTS assay, representing mitochondrial activity in living cells, also decreased when σ1R or TBK1 was depleted (Fig. 7*C*). These data indicate that MAM–TBK1 axis deficiency compromises the cellular stress response by impairing SG formation. To determine the impact of MAM–TBK1 deficiency in vivo, we treated wild-type or *Sigmar1^−/−^* mice with sodium arsenite-containing water for 3 wk (Fig. 7*D*). Motor function, as measured using rotarod tests, was significantly impaired only in arsenite-treated *Sigmar1^−/−^* mice without affecting their body weight (Fig. 7 *E* and *F*). Additionally, chronic administration of arsenite-activated TBK1 in wild-type but not in *Sigmar1^−/−^* mouse brains (Fig. 7 *G*–*I*). Furthermore, the number of C-bouton, a cholinergic synaptic terminus on motor neuron soma and a primary target of injury in ALS (36), was significantly reduced without any motor neuron degeneration (Fig. 7 *J*–*L*). Moderate activation of astrocytes was induced in arsenite-treated *Sigmar1^−/−^* mice (Fig. 7 *M*–*O*). Collectively, these findings indicate that defects in the MAM–TBK1 axis induce motor dysfunction possibly due to synaptic dysfunction on motor neurons in vivo.

## Discussion

In the current study, we demonstrate a MAM-dependent TBK1 activation mechanism (*SI Appendix,* Fig. S15). Specifically, MAM scaffolded by σ1R induced ubiquitination of nascent proteins mediated by AMFR. This ubiquitination was a key event in TBK1 activation, which is required for cellular stress tolerance due to SG formation. Moreover, TBK1 activity was impaired in sporadic ALS patients and ALS model mice. When TBK1 activation was lacking, the insufficient stress response resulted in motor dysfunction in vivo. Overall, our findings provide insights into the mechanistic link between TBK1 loss-of-function and motor neuronal dysfunction in ALS.

TBK1 haploinsufficiency is a cause of familial ALS (10, 11, 13). Several studies have revealed that TBK1 insufficiency accelerates disease onset in ALS model mice (37, 38). Importantly, we showed that TBK1 activity was also compromised in the affected tissues of sporadic ALS patients. Thus, TBK1 function is apparently crucial for motor neuronal function. Although TBK1 is well known for its role in the innate immune system and inflammation (14), its role in autophagic regulation has also been reported (15, 39). It is reasonable to suggest, therefore, that TBK1 activity, e.g., in the autophagic clearance of misfolded proteins, is associated with proteostatic stress. Indeed, TBK1 deficiency markedly increases ubiquitin-positive inclusion bodies in the motor neurons of ALS model mice (38). Nevertheless, it seems controversial that TBK1 is inactivated in ALS stress conditions. One possible interpretation is deregulation of autophagy. We have reported σ1R loss-of-function in ALS (7). In the absence of σ1R, reduced autophagosomes may prevent TBK1 translocation to the MAM where autophagosomes are formed.

We found that TBK1 activation at the MAM was dependent on AMFR-mediated polyubiquitination targeting the nascent proteins. Additionally, activated TBK1 contributed to the autophagic degradation of ribosomal subunits. Although it was not clear whether TBK1 specifically targets RPS3 and RPL26, a previous study indicated that arsenite-induced ribophagy degrades various ribosomal subunits (33), suggesting that TBK1 would also contribute to the degradation of other ribosomal subunits. AMFR is a key player in ERAD, a protein quality control mechanism active in the ER lumen (40). Interestingly, AMFR forms a complex with p97/valocin-containing protein (VCP), one of the causative gene products in ALS, to induce ERAD (41). Disruption of protein control mechanisms is considered one of the major causes of neurodegeneration in ALS. Indeed, ERAD is compromised by mutant SOD1 proteins that are causative of ALS (42). Impaired autophagy, another protein quality control mechanism, has been associated with various ALS cases related to mutations in TBK1, SQSTM1, and C9orf72 (16). In our previous studies, we demonstrated that enhanced responses to misfolded proteins via sirtuin 1 (43) or cystatin C (44, 45) ameliorated the disease progression in ALS model mice. Taken together, these findings suggest that TBK1 activity maintenance may limit translation to prevent overflow of the protein quality control mechanisms represented by ERAD.

On the other hand, TBK1 is also involved in the autophagic clearance of damaged mitochondria by mitophagy (21–23). Since the MAM contributes to the maintenance of physiological mitochondrial functions (1, 4, 5) and Sig1R prevents mitochondrial fragmentation in ALS model mice (20), it is reasonable to assume that MAM disruption impairs mitophagy. Unfortunately, in this study, we could not investigate a role of the MAM-dependent TBK1 activation in mitophagy due to a technical limitation. It remains an open question whether the MAM-dependent TBK1 activation is associated with mitophagy.

AMFR also ubiquitinates STING, a major activator of TBK1 in the innate immune response, in the presence of cytosolic double-stranded DNA (46). Furthermore, activation of the STING–TBK1 axis by endogenous ligands is reportedly involved in ALS pathogenesis (47). We cannot exclude the possibility that AMFR is also involved in the STING-mediated TBK1 activation; however, MAM was not associated with TBK1 activation via TLR ligands, TLR suppression, or STING suppression, suggesting that MAM-dependent TBK1 activation is independent of the STING-associated innate immune response. However, our observations do not exclude the possibility that TBK1 is activated in vivo via innate immune pathways. Future studies are required to determine the involvement of the innate immune system.

Studies, including ours, have provided evidence that MAM disruption is a common pathomechanism in ALS (7, 48, 49). For example, MAM disruption is induced by various familial ALS causative genes (9), aberrant σ1R aggregation occurs in sporadic ALS patients (50), and MAMs with VAPB, another tethering factor at the MAM, are compromised in ALS (51, 52). Thus, impairment of the physiological functions of the MAM is closely associated with ALS pathomechanisms. The MAM has various physiological functions, including Ca^2+^ transfer to mitochondria, lipid synthesis, and glucose homeostasis (1, 2). In particular, the MAM is known to be involved in proteostasis, represented by ER stress response (53) and autophagosome formation (54). In our study, AMFR ubiquitinated nascent proteins at the MAM and recruited autophagic factors, including TBK1 and SQSTM1/p62, into the MAM; the subsequent TBK1 activation contributed to the degradation of ribosomal subunits via autophagy. Molecular chaperones, such as σ1R, calnexin, calreticulin, and glucose-regulated protein-78/BiP, are known to localize at the MAM. Notably, σ1R and VAPB localize at the MAM and regulate autophagy (55, 56). Mutant SOD1 species accumulate at the MAM (7), and AMFR and VCP are the crucial components of ERAD (38). Taken together, these observations suggest that the MAM is a center for proteostasis, and its disruption may cause an impaired stress response, leading to motor neuron vulnerability. Therefore, preventing MAM disruption could improve proteostasis and contribute to future therapeutic strategies for ALS. We and others have shown that the administration of synthesized σ1R agonists improved the pathology of ALS model mice (7, 57). We postulate that σ1R activation may be a possible means to realize such therapeutic strategies.

On the other hand, MAM disruption did not seem to be a common pathology in neurodegenerative diseases. In Alzheimer’s disease (AD), excess MAM contributes to the production of amyloid-β via the accumulation of presenilin-1, an enzymatic complex processing amyloid-β (58). Although both excess and deficiency of the MAM result in neurodegeneration, there are differences in affected neurons, phenotypes, and accumulated pathological proteins. Indeed, type 2 diabetes or obesity is a risk factor for AD, whereas it is known to reduce the risk of developing ALS (59). In obesity, increased MAM activity leads to insulin resistance (3). These observations suggest the possibility that deregulation of the MAM compromises cellular homeostasis to induce neurodegeneration independent of MAM levels. In future studies, it would be necessary to elucidate a precise mechanism of how the MAM is deregulated in each disease.

SGs are stress-induced inclusions composed of RNA and RNA-binding proteins (36). SGs contribute to reducing proteotoxicity under stress conditions by preventing protein synthesis. We showed that SG formation was delayed when TBK1 activity was inhibited, resulting in an increased vulnerability, both in vitro and in vivo. Consistent with our findings, autophagic inhibition is known to delay SG formation via insufficient degradation of translational complexes (35). Hence, TBK1-mediated autophagy seems to be required for rapid SG formation and is crucial for achieving proteotoxic tolerance. Moreover, aberrant SG formation has been suggested as an ALS pathomechanism (60). TAR DNA-binding protein 43 (TDP-43), a protein ubiquitously accumulated in ALS, is localized at SGs, and demixing TDP-43 without its sequestration into SGs compromises the physiological function of TDP-43, leading to ALS pathology (61). Another ALS-related RNA-binding protein, fused-in-sarcoma (FUS), is also known to be localized at SGs (62). Mutations in *TIA1*, a recently identified ALS causative gene, also compromise SGs to induce neurodegeneration (63). Although it is unclear whether the delayed SG formation observed in the present study similarly affected these ALS-related proteins, abnormalities in SGs are intrinsically associated with the pathogenesis of ALS.

Finally, our findings emphasize the importance of organelle contact sites in cellular homeostasis. Although our study has the limitation of lacking a direct link between MAM-dependent TBK1 insufficiency and ALS pathogenesis, TBK1 impairment associated with MAM alternation would be a part of the general pathomechanisms in cellular proteostatic stress response. Indeed, MAM–TBK1 deficiency was associated with motor dysfunction possibly due to a synaptic dysfunction in vivo. Accumulating evidence indicates that organelle contact sites, including the MAM, play essential roles in the physiological maintenance of cellular components (64). Multiple physiological functions of the MAM may explain the diversity of cytotoxicity in ALS, including proteostatic stress. Therefore, targeting the MAM has a therapeutic potential for ALS. It is probable that organelle contact sites other than the MAM are also involved in ALS pathology. Moreover, MAM deregulation has also been reported in other neurodegenerative diseases, including Alzheimer’s disease (65), Parkinson’s disease (66), and Huntington’s disease (67). Continued research aimed at identifying the precise mechanisms underlying the functions of organelle contact sites is important to the future development of therapeutic strategies for such neurodegenerative diseases.

## Materials and Methods

For detailed methods and antibody list used in this study, see *SI Appendix*.

### Postmortem Human Tissues.

Frozen brain (frontotemporal cortices) or spinal cord samples from four cases with sporadic ALS or ALS with frontotemporal lobar degeneration (ALS/FTLD) and four other neurological disease patients as controls were obtained by autopsy with informed consent (*SI Appendix*, Table S2). The diagnosis of ALS was confirmed by El Escorial diagnostic criteria defined by the World Federation of Neurology. The ethics committee of Nagoya University approved the collection of tissues and their use in this study. Brain tissues were immediately frozen in liquid nitrogen and stored at −80 °C until use.

### Animals.

For detailed information on the animals used in this study, see *SI Appendix*. The experiments using genetically modified animals and organisms were approved by the Animal Care and Use Committee and recombinant DNA experiment committee of Nagoya University.

### Cell Culture, Plasmids, siRNAs, and Transfection.

HeLa (#CCL-2) and SH-SY5Y (#CRL-2266) cells were obtained from American Type Culture Collection (ATCC). SH-SY5Y cells were differentiated by adding 10 µM all-trans retinoic acid (Sigma-Aldrich Inc.) before the day of transfection. MEFs were established from E14 embryos of B6J or *Sigmar1^−/−^* mice, as described in *SI Appendix*. siRNAs were purchased from Integrated DNA Technologies Inc.). For information on the plasmids and transfection, see *SI Appendix*.

### Isolation of MAM, Immunofluorescence, Coimmunoprecipitation, and Immunoblotting.

Isolation of the MAM and immunofluorescence were performed as described previously (7). Detailed methods are described in *SI Appendix*.

### Proximal Labeling and Mass Spectrometry (MS) Analysis.

The proximal labeling assay using σ1R-APEX2 and MS analysis were performed as described in *SI Appendix*.

### In Vitro and In Vivo Arsenite Toxicity Assay.

HeLa cell viability was evaluated using Hoechst 33342 (Sigma-Aldrich) or MTS assay (Promega Corporation) after treatment with 0.1 mM arsenite overnight. The protocols for oral administration of arsenite are described in *SI Appendix*.

### Statistics.

All semiquantitative immunoblotting data, cell viability data, score of rotarod test, and mouse body weight were analyzed by one-way ANOVA followed by multiple comparison tests with Sidak’s correction for three or more groups and Welch’s t-tests for two groups. Time course of SG formation was analyzed by two-way ANOVA following Tukey’s multiple comparison tests. All statistical analyses were performed using GraphPad Prism software (GraphPad Software). No randomization or blinding was used in this study.

## Supplementary Material

Appendix 01 (PDF)

Dataset S01 (XLSX)

## Data Availability

All study data are included in the article and/or supporting information.
