# Supplementary material for: Mitochondria-associated membrane collapse impairs TBK1-mediated proteostatic stress response in ALS
Source: Proc Natl Acad Sci U S A. 2023 Nov 15;120(47):e2315347120. doi: 10.1073/pnas.2315347120 (PMC10666035; doi:10.1073/pnas.2315347120)
Supplement: Supplementary file 1 — Appendix 01 (PDF) [file pnas.2315347120.sapp.pdf]

PNAS

Supplementary Information for Mitochondria-associated membrane collapse impairs TBK1-mediated proteostatic stress response in ALS

Seiji Watanabe[a], Yuri Murata[a], Yasuyoshi Oka[b], Kotaro Oiwa[a,c], Mai Horiuchi[a], Yohei Iguchi[c], Okiru Komine[a], Akira Sobue[a,d], Masahisa Katsuno[c,e], Tomoo Ogi[b,e], *Koji Yamanaka[a,e,f]

[a]Department of Neuroscience and Pathobiology, Research Institute of Environmental Medicine, Nagoya University, Nagoya, Aichi, Japan; [b]Department of Genetics, Research Institute of Environmental Medicine, Nagoya University, Aichi, Japan; [c]Department of Neurology, Nagoya University Graduate School of Medicine, Nagoya, Aichi, Japan; [d]Medical Interactive Research and Academia Industry Collaboration Center, Research Institute of Environmental Medicine, Nagoya University, Aichi, Japan; [e]Institute for Glyco-core Research (iGCORE), Nagoya University, Aichi, Japan; [f]Center for One Medicine Innovative Translational Research (COMIT), Nagoya University, Nagoya, Aichi, Japan

*Corresponding authors: Koji Yamanaka and Seiji Watanabe

Email: koji.yamanaka@riem.nagoya-u.ac.jp (KY), swtnb@riem.nagoya-u.ac.jp (SW)

This PDF file includes:

Supplementary Materials and Methods
Figures S1 to S15
Tables S1 and S2
SI References

Supplementary Materials and Methods

Animals. Transgenic mice expressing mutant human SOD1 (B6.Cg-Tg(SOD1*G85R) 148Dwc/J, B6.Cg-Tg(SOD1*G93A) 1Gur/J) were obtained from the Jackson Laboratory (Bar Harbor, ME, USA) or were gifts from Dr. Don Cleveland (University of California, San Diego). *Sigmar1*$^{-/-}$ mice (B6.129S5-Sigmar1Gt(OST422756)Lex/Mmucd) were obtained from the Mutant Mouse Regional Resource Center (MMRRC, University of California, Davis). C57BL/6J (B6J) mice were obtained from CLEA Japan Inc. (Tokyo, Japan). *Tbk1* knockout (*Tbk1*$^{-/-}$) mice were established by backcrossing *Tbk1*, *IKBKE*, and *Tnf* triple knock-out mice (#nbio156) obtained from Laboratory Animal Resource Bank, National Institute of Biomedical Innovation, Health, and Nutrition, Japan to B6J. *Tnf* deficiency (1) was retained in *Tbk1*$^{-/-}$ mice to avoid embryonic lethality. Genotyping of mice was performed as described previously (2) or according to the instructions from the providers. All the mice were maintained under a standard specific pathogen-free environment (12 h light-dark-cycle; 23 ± 1 ºC; 50 ± 5 % humidity) with free access to food and water. Mice were treated in compliance with the guidelines established by the Institutional Animal Care and Use Committee of Nagoya University.

Cell culture, plasmids, and transfection. Wild-type or *Tbk1* knockout MEFs were established from E14 embryos of *Tbk1*$^{-/-}$ mice using the same procedure described elsewhere (3). HeLa cells and Neuro2a cells were maintained in high-glucose (4.5 g/L) Dulbecco's Modified Eagle's Medium (DMEM; #11965) supplemented with 10 %(v/v) fetal bovine serum (FBS), penicillin (100 U/mL), and streptomycin (100 U/mL) (all from Thermo Fisher Scientific Inc., Waltham, MA, USA). SH-SY5Y (#CRL-2266) cells were maintained in a 1:1 mixture of DMEM and Ham's F-12 (DMEM/F12) (#113200, Thermo Fisher Scientific) supplemented with 10 %(v/v) FBS, penicillin, and streptomycin. MEFs were maintained in high-glucose DMEM supplemented with 10 %(v/v) FBS, penicillin, and streptomycin.

Human TLR3 expression plasmid; pUNO1-hTLR3 (#puno1-htlr3) and human TLR4/MD2 dual expression plasmid; pDUO-hMD2/TLR4 (#pduo-mmd2tlr4) were purchased from InvivoGen. Human σ1R, TBK1, or AMFR cDNA was cloned with FLAG, c-myc, or influenza hemagglutinin (HA) tag, respectively, from total RNA of HeLa cells into pcDNA3.1(+) (Thermo Fisher Scientific) using Seamless Ligation Cloning Extract (SLiCE) from Escherichia coli HST02 (4) and the canonical sequences were confirmed. APEX2 cDNA (5) tagged with V5 tag was synthesized by gBlocks Gene Fragments service (Integrated DNA Technologies Inc. (IDT), Coralville, IA, USA) and replaced with a FLAG tag at the C-terminal of σ1R.

Predesigned 27mer Dicer-Substrate Short Interfering RNAs (siRNAs) specific for human or mouse genes were obtained from IDT. Transfection was carried out using Lipofectamine 2000 and RNAiMAX (both from Thermo Fisher Scientific) according to the manufacturer's instructions. For MAM isolation, cells were transfected using linear polyethyleneimine (MW 25,000) (PEI) (#239666, Polysciences Inc., Warrington, PA, USA). Briefly, 36 µg/dish plasmids dissolved in 2.9 mL/dish Opti-MEM I (#31985, Thermo Fisher Scientific) was mixed with 109 µL PEI (1.0 mg/mL). The mixture was incubated for 15 min at room temperature, then added to the cells seeded on a 15 cm cell culture dish. The cells were incubated overnight before MAM isolation.

Antibodies and reagents. Primary antibodies used in this study are listed in Table S1. Alexa Fluor-conjugated and horseradish peroxidase (HRP)-conjugated secondary antibodies were purchased from Thermo Fisher Scientific and Jackson ImmnoResearch Laboratories, Inc. (West Grove, PA, USA). HRP-conjugated streptavidin (#21130) was obtained from Thermo Fisher Scientific. LPS from Escherichia coli 055:B5 (LPS; #L2880) and sodium arsenite (#S7400) were purchased from Sigma-Aldrich. Poly I:C (#tlrl-pic) and amlexanox (#inh-amx) were purchased from InvivoGen (San Diego, CA, USA). Tunicamycin (#202-08241) and CHX (#037-20991) were purchased from FUJIFILM Wako Pure Chemical Corporation (Osaka, Japan). MG-132 (#3175-v) was purchased from Peptide Institute Inc. (Osaka, Japan).

Immunofluorescence. Mice at the indicated age were deeply anesthetized and transcardially perfused with phosphate-buffered saline (PBS) following 4 %(w/v) paraformaldehyde in 0.1 M phosphate buffer for 10 minutes, respectively. After incubation with 30 %(w/v) sucrose in PBS,

dissected lumbar spinal cords were embedded in Tissue-Tek OCT compound medium (Sakura Finetek, Tokyo, Japan) and frozen at −80 ºC until use. For σ1R staining, sections or cells on slide chambers (LabTek II from Thermo Fisher Scientific) were incubated in 10 mM Tris-HCl (pH 9.5) and 6 M urea at 85 ºC for 10 min (6). After blocking, 12 µm-sliced spinal cord sections were incubated with primary antibodies overnight at 4 ºC. Bound primary antibodies were detected with Alexa Fluor 488-conjugated anti-mouse or Alexa Fluor 546-conjugated anti-goat IgG secondary antibodies (both used in 1:1000; Thermo Fisher Scientific). Images were obtained by confocal laser scanning microscopy (LSM-700; Carl Zeiss AG, Oberkochen, Germany) and the equipped software (Zen; Carl Zeiss).

Paraquat treatment of Neuro2a cells. Neuro2a cells were seeded at 5.0×10^4 /well on 4 well chamber slides (Lab Tek II) coated with poly-D-lysine (Sigma-Aldrich). On the next day, the medium was changed to DMEM containing 2 %(v/v) FBS and 2.5 mM dibutyryl adenosine 3',5'-cyclic monophosphate sodium salt (Nacalai Tesque, Kyoto, Japan) with or without 1 mM paraquat (Sigma-Aldrich). The cells were incubated for 24 h, fixed by 4 %(w/v) paraformaldehyde in 0.1 M phosphate buffer for 20 minutes, then subjected to the immunofluorescence using anti-G3BP antibody.

Co-immunoprecipitation and immunoblotting. Protein concentration in each fraction was measured using a Bio-Rad Bradford protein assay kit (Bio-rad Laboratories Inc., Richmond, CA, USA). For co-immunoprecipitation, HeLa cells were seeded at 4.0×10^5 cells/well on 6-well plates. After transfection, the cells were incubated overnight and lysed in 0.5 mL ice-cold RIPA buffer [50 mM Tris-HCl (pH 7.4), 150 mM NaCl, 1 %(v/v) Nonidet P-40 (Sigma-Aldrich), 0.5 %(w/v) sodium deoxycholate (Wako)] supplemented with cOmplete protease inhibitor and PhosSTOP (both from Roche), and incubated with anti-FLAG or anti-polyubiquitin antibodies overnight at 4 ºC with gentle agitation, followed by incubation with Dynabeads M-280 Sheep anti-mouse IgG (Thermo Fisher Scientific) for further 1.5 h. The beads were washed four times with RIPA buffer. Proteins were eluted by incubation in 50 µL of 2×sodium dodecyl sulfate (SDS)-polyacrylamide gel electrophoresis (PAGE) loading buffer [125 mM Tris-HCl (pH 6.8), 4 %(w/v) SDS, 10 %(v/v) glycerol, 0.04 %(w/v) bromophenol blue, 2.5 %(v/v) 2-mercaptoethanol] for 3 min at 95 ºC.
An equal amount (15 µg/lane for whole cell or tissue lysates, 5 µg/lane for the isolated fractions) or an equal volume (10 µL/lane for coimmunoprecipitation) was subjected to SDS-PAGE and transferred on an immobilon-P membrane (EMD Millipore). The membrane was blocked with blocking buffer [50 mM Tris-HCl (pH 8.0), 150 mM NaCl, 0.05 %(v/v) Tween-20, and 2 %(w/v) bovine serum albumin (Wako)], followed by incubation with primary and secondary antibodies. Images were obtained with Immobilon Crescendo Western HRP Substrate (EMD Millipore, Burlington, MA, USA) using LAS-4000 mini (Cytiva, Tokyo, Japan) with the equipped software (Multi-Gauge; Cytiva).

Proximal labeling and MS analysis. SH-SY5Y cells transiently expressing σ1R-APEX2 were incubated for 30 min in a serum-free DMEM containing 0.5 mM biotin tyramide (Iris Biotech GmbH, Marktredwitz, Germany). After the incubation, cells were treated with 0.1 mM H_2O_2 for 1 min at room temperature. The labeling reaction was quenched by washing the cells with ice-cold PBS supplemented with 0.5 mM Trolox and 10 mM sodium ascorbate (both from Tokyo Chemical Industry Co., Ltd., Tokyo, Japan) three times. Cells were homogenized in ice-cold 10 mM HEPES-KOH (pH 7.4), 250 mM sucrose supplemented with cOmplete protease inhibitors. The homogenates were centrifuged for 5 min at 600×g, 4 ºC. The supernatant was further centrifuged for 20 min at 10,300×g, 4 ºC. The pellet (crude mitochondria) was resuspended in ice-cold RIPA buffer and purified using Dynabeads M-280 streptavidin (Thermo Fisher Scientific). The purified proteins were eluted by boiling for 5 min in a 2×SDS-PAGE loading buffer containing 3 M biotin and 20 mM dithiothreitol (DTT). The eluted samples were resolved by SDS-PAGE, and the gels were stained with Negative Gel Stain MS Kit (FUJIFILM). The gels were cut into pieces, and proteins were in-gel digested. Briefly, the gel slice was desiccated with acetonitrile, treated with 10 mM dithiothreitol (Thermo Fisher Scientific) for 30 min at 56 ºC and then with 25 mM iodoacetamide (Merck Millipore) for 30 min in the dark at room temperature. Proteins were in-gel digested with Trypsin/Lys-C Mix (Promega) overnight at 37 ºC. Digested peptides were analyzed on a Q Exactive

Orbitrap mass spectrometer (Thermo Fisher Scientific) equipped with an EASY-Spray ion source (Thermo Fisher Scientific) and coupled to an EASY nano-LC 1000 system (Thermo Fisher Scientific) using an EASY-Spray column (50 cm × 75 μm inner diameter, 2 μm particles; Thermo Fisher Scientific) with a 60-minute linear gradient from 5 % to 35 % acetonitrile and 0.1 % formic acid at a flow rate of 300 nL/min. The washout followed at 95 % acetonitrile in 0.1 % formic acid for 10 min. Spray voltage was set to 2.0 kV, s-lens RF level at 50, and heated capillary temperature 250 ºC. All experiments were performed in the data-dependent acquisition mode to automatically isolate and fragment top10 multiply-charged precursors (+2, +3, and +4) according to their intensities. Former target ions were dynamically for 15 seconds excluded, and all experiments were acquired using positive polarity mode. Full scan resolution was set to 70,000, and the mass range was set to m/z 380-1500. Full scan ion target value was 3×10^6 allowing a maximum fill time of 60ms. Higher-energy collisional dissociation (HCD) fragment scans were acquired with the optimal setting for parallel acquisition using 2.0 m/z isolation width and normalized collision energy of 27.

MS raw data processing and analysis. The data were analyzed using SEQUEST tool in Proteome Discoverer 2.1 (Thermo Fisher Scientific) and searched in the complete human proteome database (Swiss-Prot, SIB). The mass tolerances for the precursor and fragment were 10 ppm and 0.02 Da, respectively. Cysteine carbamidomethylation was included as a fixed modification and N-terminal protein acetylation and methionine oxidation were included as variable modifications. Peptide identification was filtered at a false discovery rate (FDR) < 1 %.

Oral administarion of arsenite. We modified the protocols reported in rats (7). Briefly, C57BL/6J-Jcl or *Sigmar1*$^{-/-}$ mice were randomly divided into two groups that fed water with or without 30 μg/mL sodium arsenite ad libitum. The treatment was started at 10 months of age and continued for 3 weeks. After the treatment, a rotarod test was performed as previously described (8).

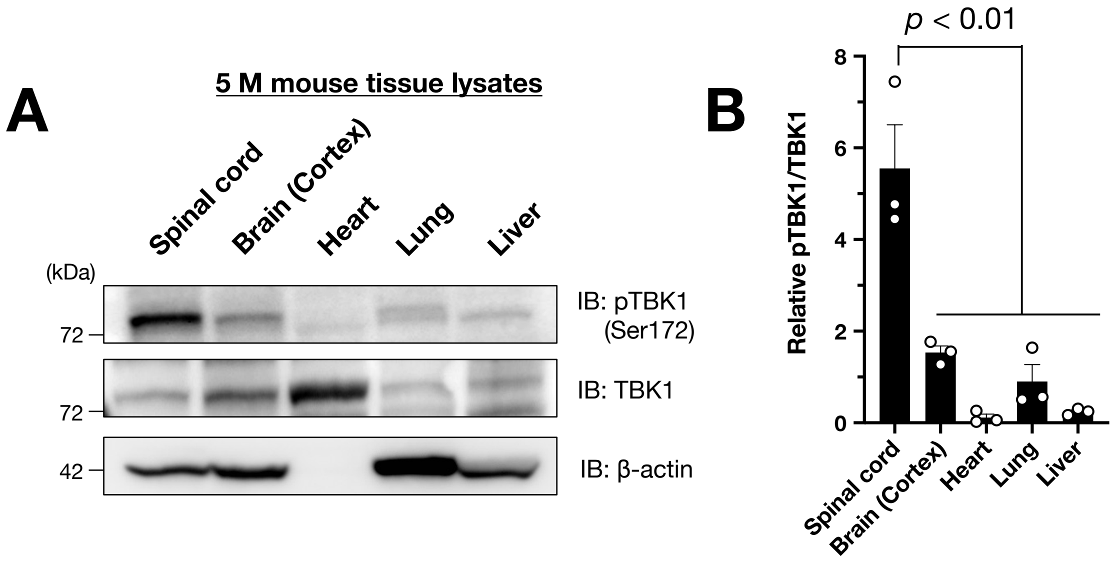

Fig. S1. TBK1 activity in various mouse tissues. Tissues from 5 months old (5 M) C57BL/6J mice were lysed in RIPA buffer, and 20 μg of total proteins per lane were subjected to immunoblotting. Representative immunoblotting images are shown in (A). Relative pTBK1 levels normalized to total TBK1 were plotted in B. The data are expressed as mean ± SEM, analyzed by one-way ANOVA following post-hoc Tukey's multiple comparison tests, and the *p*-values are shown as the numbers.

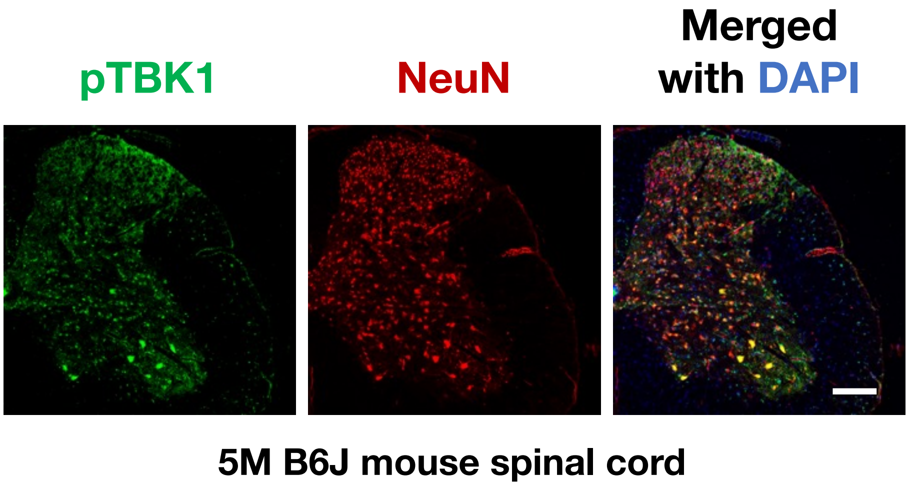

Fig. S2. TBK1 is predominantly activated in neurons in the spinal cords of wild-type (B6J) mice. Sections of the mouse spinal cords were immunostained using anti-pTBK1 and anti-NeuN antibodies. Although pTBK1 signal was observed in most neurons, including those around the central canal or in the dorsal horn, motor neurons in the ventral horn showed the highest fluorescence intensity among all neurons.

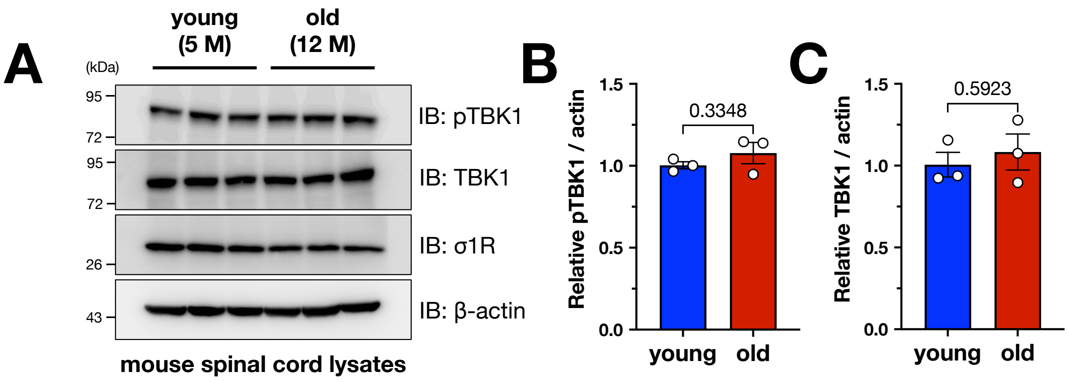

Fig. S3. TBK1 activity in young (5 months old; 5 M) or old (12 months old; 12 M) mouse spinal cords. Spinal cords from C57BL/6J mice were lysed in RIPA buffer, and 20 µg/lane of total proteins were subjected to immunoblotting. Representative immunoblotting images are shown in (A). Relative levels of quantified pTBK1 or total TBK1 were plotted in (B) and (C), respectively. The data are expressed as mean ± SEM, analyzed by Student's t-tests, and the *p*-values are shown as numbers.

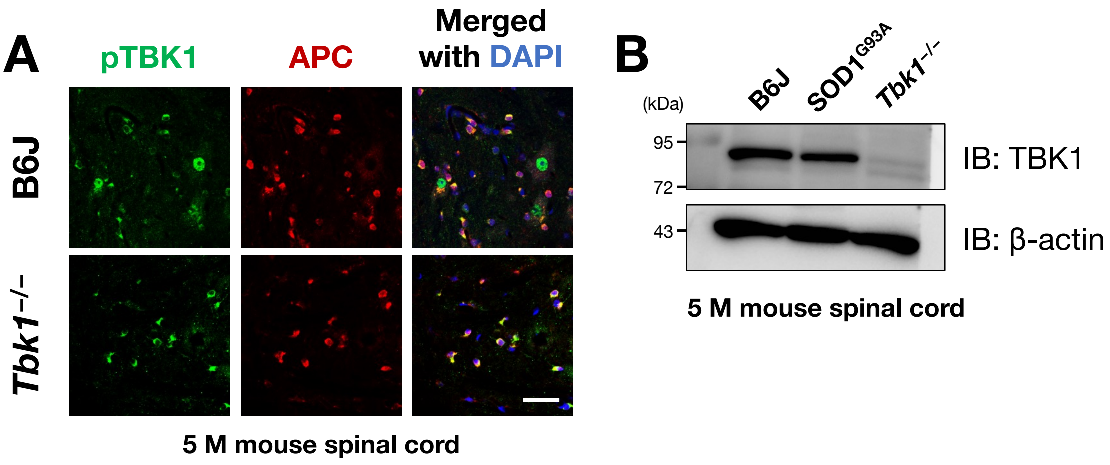

Fig. S4. Non-specific immunoreactivity of pTBK1 antibody in oligodendrocytes. Representative immunohistochemical images of the wild-type (B6J) or *Tbk1* knockout (*Tbk1*$^{-/-}$) mouse spinal cords are shown in (A). *Tbk1* deficiency was confirmed by immunoblotting shown in (B). Note that non-neuronal pTBK1 puncta, which co-localized with oligodendrocyte marker APC, remained in *Tbk1*$^{-/-}$ mice. Scale bar: 10 μm.

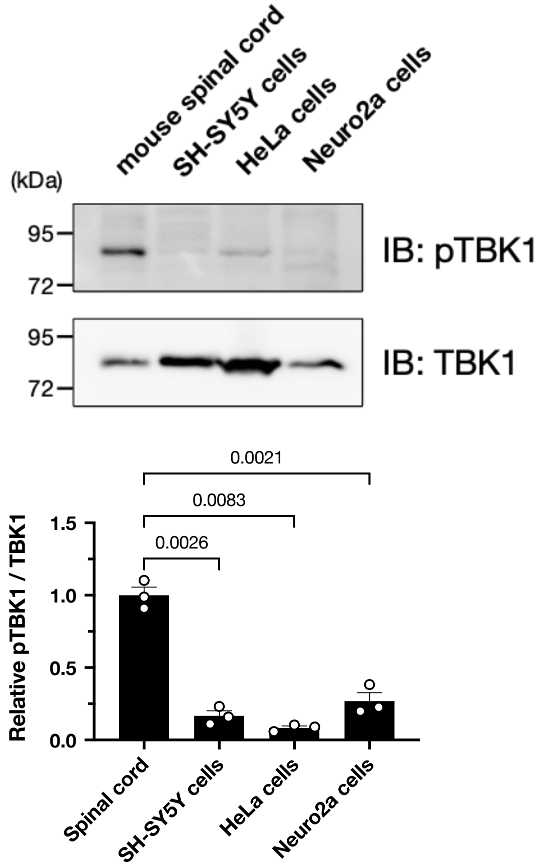

Fig. S5. TBK1 is inactivated in cultured mammalian cell lines. Mouse spinal cord or whole cell lysates extracted with RIPA buffer (15 μg/lane) were subjected to immunoblotting. Data from three independent experiments were plotted as mean ± SEM in the bottom panel and analyzed using one-way ANOVA following Tukey's multiple comparison tests, and the *p*-values are shown as numbers.

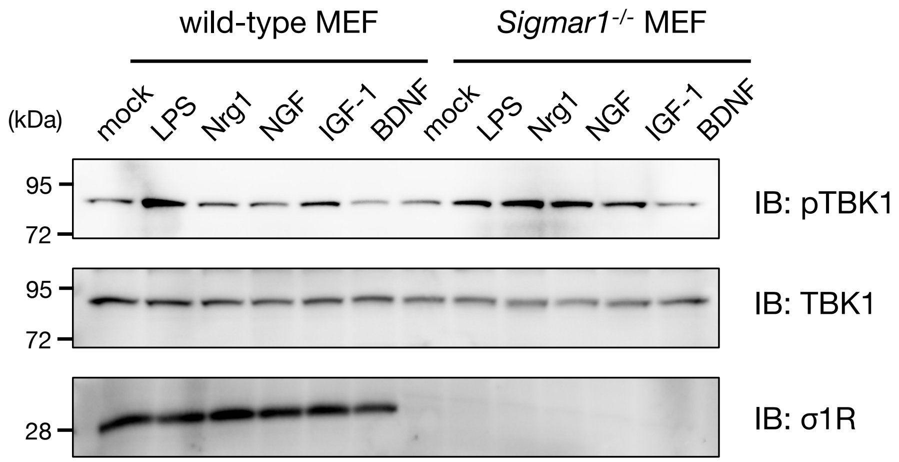

Fig. S6. Trophic factors did not activate TBK1 dependent on MAM. The wild-type or Sigmar1$^{-/-}$ MEF were treated with lipopolysaccharide (LPS; 1μg/mL), murine neuregulin-1 (Nrg1; #9875-NR from R&D Systems, Inc., Minneapolis, MN, USA), murine nerve growth factor (NGF; #354005 from BD biosciences, Franklin Lakes, NJ, USA), human insulin-like growth factor-1 (IGF-1; #100-11 from Peprotech US, Cranbury, NJ, USA), or human/murine/rat brain-derived neurotrophic factor (BDNF; #420-00 from Peprotech) (all 0.2 μg/mL) for overnight. The cells were lysed in RIPA buffer supplemented with protease inhibitor and PhosSTOP and subjected to immunoblotting.

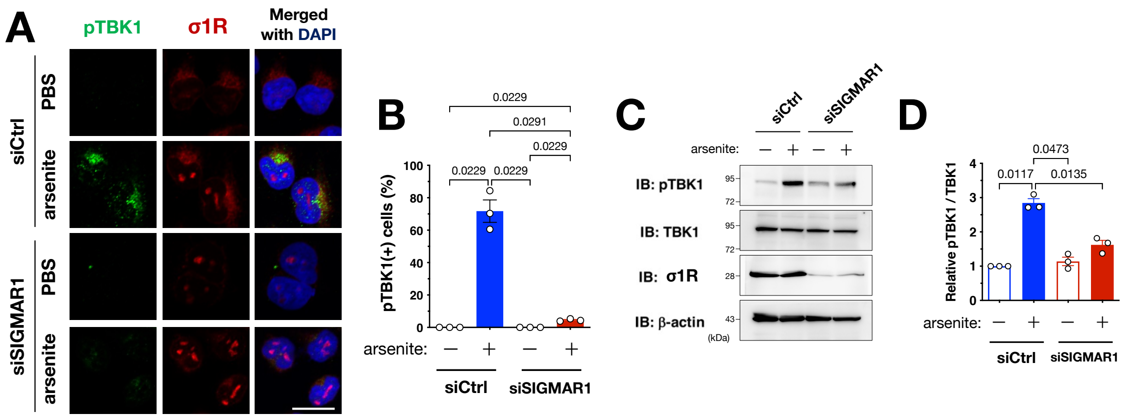

Fig. S7 Activation of TBK1 by arsenite treatment (0.1 mM, 30 min) requires σ1R in HeLa cells. The cells were treated with siRNAs against SIGMAR1 (siSIGMAR1) one day before the arsenite treatment. Representative immunocytochemical (A) and immunoblotting (C) images are shown with quantification of relative pTBK1-positive cell numbers or protein levels in (B) and (D), respectively. The data are expressed as means ± SEM; *p*-values are shown. Scale bar: 10 μm.

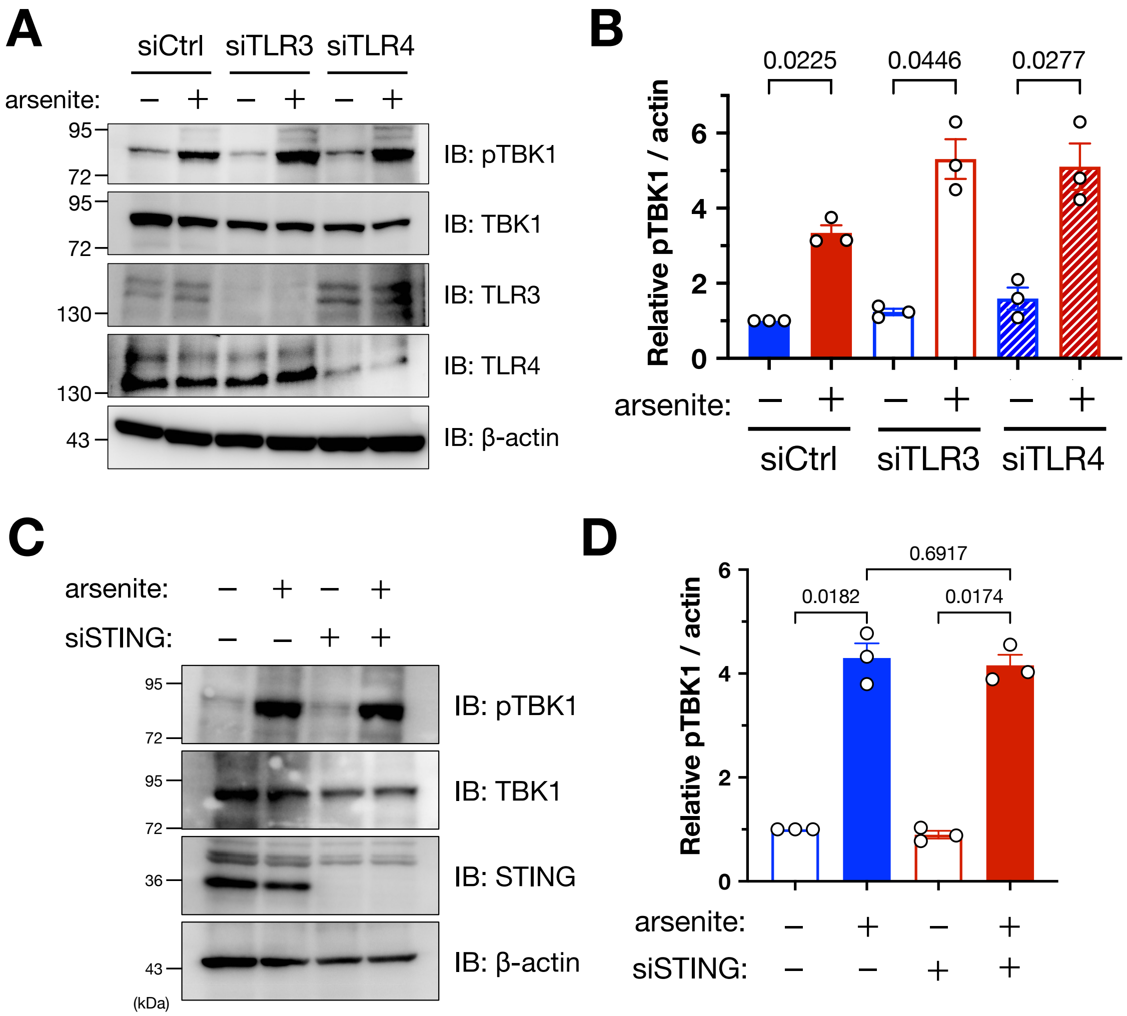

Fig. S8. TLR3, TLR4, and STING, major activators of TBK1, are independent of MAM-dependent TBK1 activation under proteostatic stress conditions. HeLa cells transfected with siRNA against *TLR3*, *TLR4*, or *STING* gene were subjected to arsenite treatment (0.1 mM, 30 min). After the treatment, pTBK1 levels were measured by immunoblotting. Representative immunoblotting images are shown in (A) and (C). Quantifications of relative pTBK1 levels are shown in (B) and (D). The data replicated in three independent experiments are expressed as mean ± SEM, analyzed by one-way ANOVA following post-hoc Tukey's multiple comparison tests, and the *p*-values are shown as numbers.

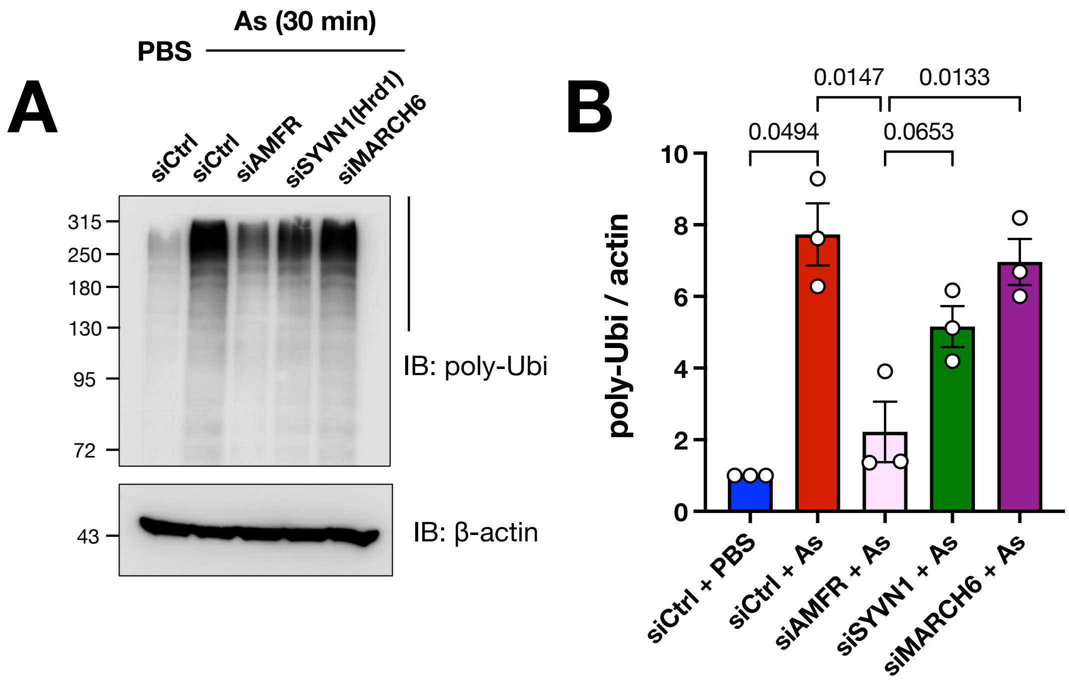

Fig. S9. AMFR predominantly mediates ubiquitination under arsenite treatment. HeLa cells transfected with siRNA against three major E3 ubiquitin ligase related to ER-associated degradation (ERAD), AMFR, SYVN1(Hrd1), or MARCH6, were treated with 0.1 mM arsenite for 30 min (As). The cells were lysed in an SDS-loading buffer and subjected to immunoblotting. The representative immunoblotting images are shown in (A), and the quantified relative poly-ubiquitinated proteins (poly-Ubi) normalized to β-actin in the three independent experiments were plotted as mean with SEM in (B). The black bar in (A) indicates the region used for the quantification. The data were analyzed by one-way ANOVA following Tukey's multiple comparison tests, and *p*-values are shown as the numbers in (B).

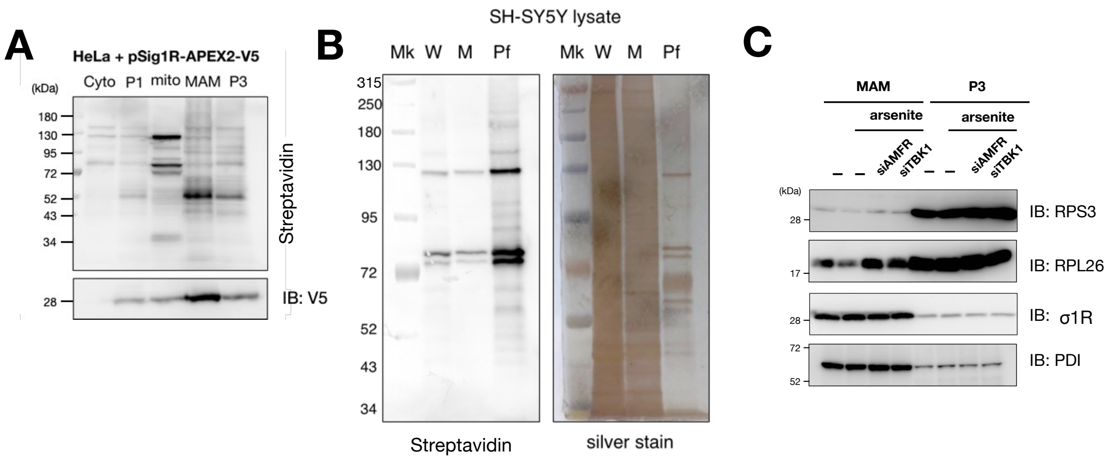

Fig. S10 A proximity labeling assay using σ 1-APEX revealed the involvement of ribosomal proteins at the MAM. (A) σ1-APEX predominantly localized at the MAM and specifically labeled MAM-related proteins. MAM- or mitochondria-specific labeling was confirmed by HRP conjugated streptavidin in fractionated HeLa cells. (B) SHSY-5Y cell lysates purified using streptavidin magnetic beads were analyzed. Mk, molecular weight marker; W, whole lysates; M, crude mitochondria; Pf, proteins purified by streptavidin. (C) Depletion of AMFR or TBK1 prevented ribosomal subunit degradation at the MAM following arsenite treatment. Hela cells treated with siAMFR or siTBK1, before arsenite treatment, then MAM and P3 fractions were analyzed by immunoblotting.

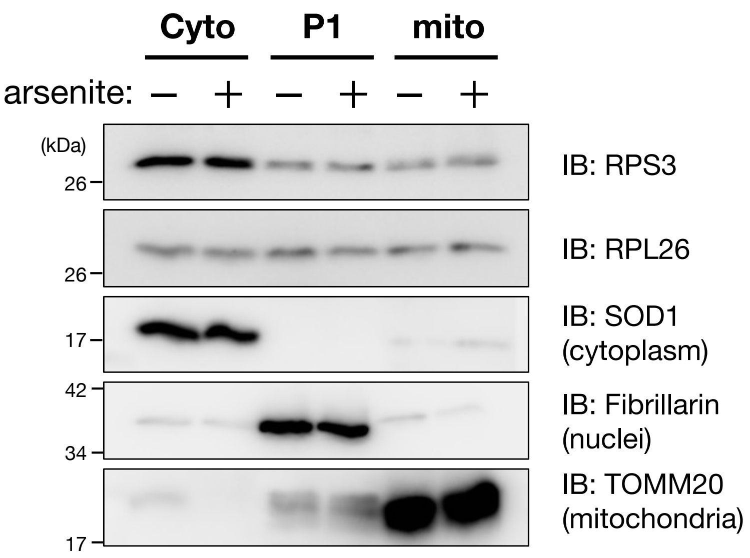

Fig. S11. Ribosomal subunits, RPS3 and RPL26, were not translocated to the non-MAM fractions after arsenite treatment. HeLa cells were fractionated after arsenite treatment (0.1 mM, 30 min), and the fractions except for the MAM and P3 were analyzed by immunoblotting. Note that the amounts of RPS3 or RPL26 in each fraction were unaffected by the arsenite treatment, suggesting that their reduction in the MAM fraction is dependent on degradation rather than translocation into the other fractions.

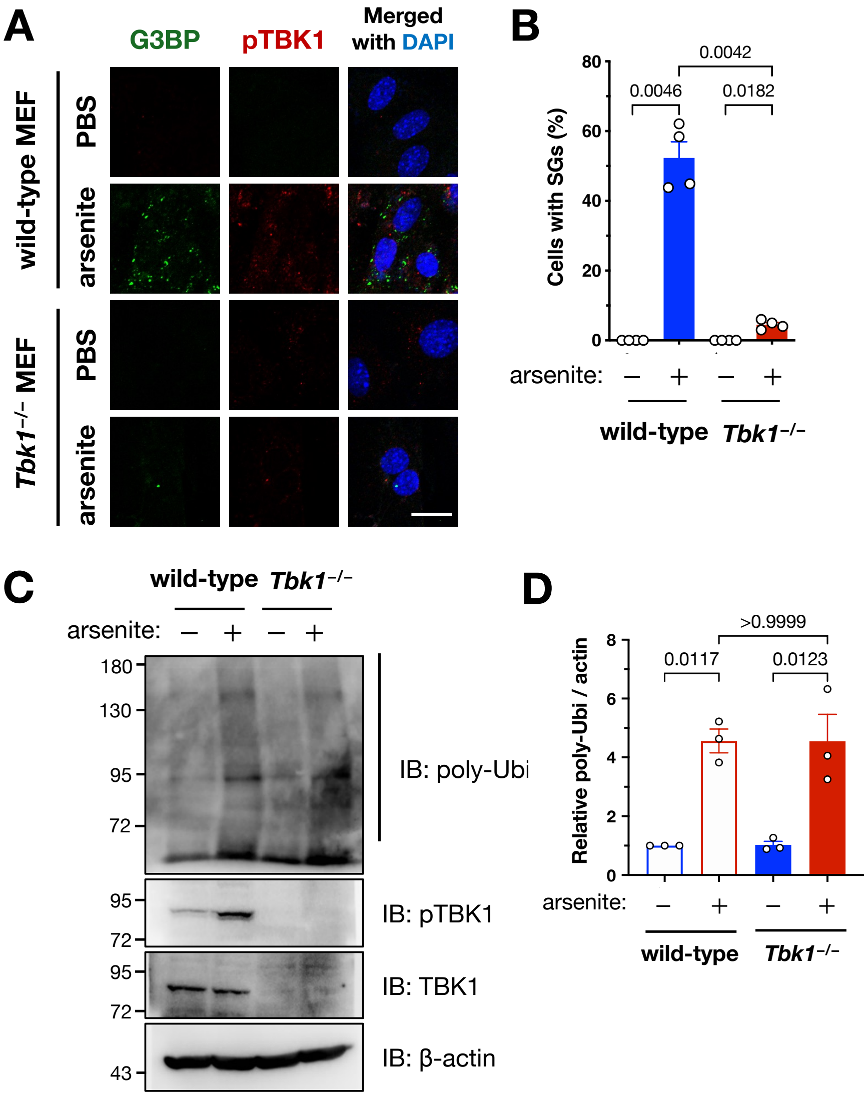

Fig. S12. SG formation was delayed without affecting poly-ubiquitin accumulation in *Tbk1*$^{-/-}$ MEFs. SGs were induced by arsenite treatment (0.1 mM, 30 min) and visualized by G3BP. Representative immunocytochemical images are shown in (A), and the number of SGs was quantified in (B). Immunoblotting analyses were also performed to confirm TBK1 deficiency in *Tbk1*$^{-/-}$ MEFs (C and D). Note that poly-ubiquitin accumulation after arsenite treatment was unaffected in *Tbk1*$^{-/-}$ MEFs, suggesting that TBK1 is involved downstream of poly-ubiquitination. The data are expressed as mean ± SEM, analyzed by one-way ANOVA following Tukey's multiple comparison tests, and *p*-values are shown as numbers. Scale bar: 20 μm.

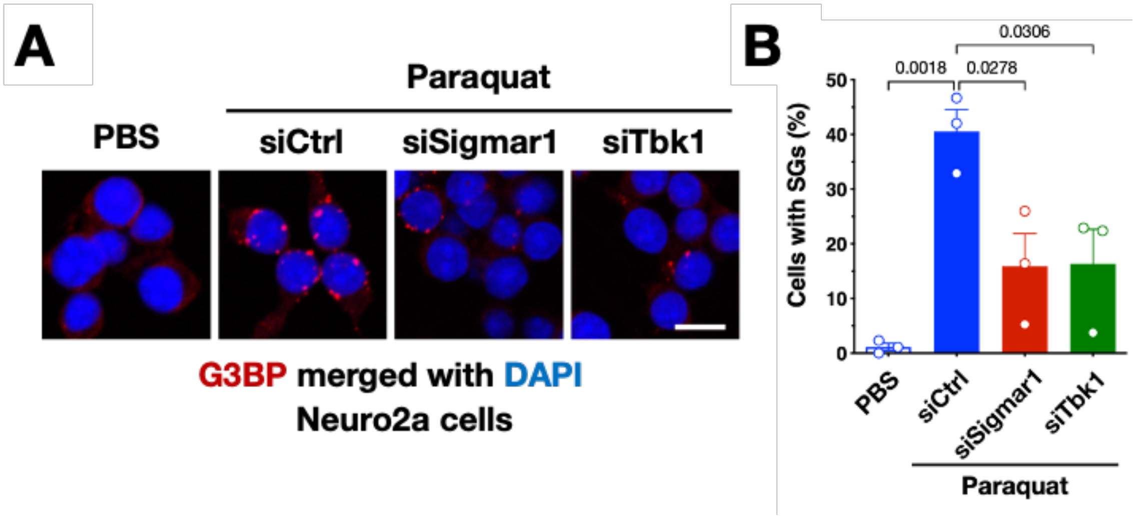

Fig. S13. Suppression of σ1R or TBK1 prevented SGs' formation in mouse neuroblastoma Neuro2a cells. Neuro2a cells were treated with 1 mM Paraquat for 24 hours. The cells were immunostained with anti-G3BP antibody. Representative fluorescent images are shown in (A), and the percentage of the cells with SGs are quantified in (B), respectively. The data are expressed as mean ± SEM, analyzed by Student's t-tests (B) and *p*-values are shown as the numbers. Scale bar: 10 μm.

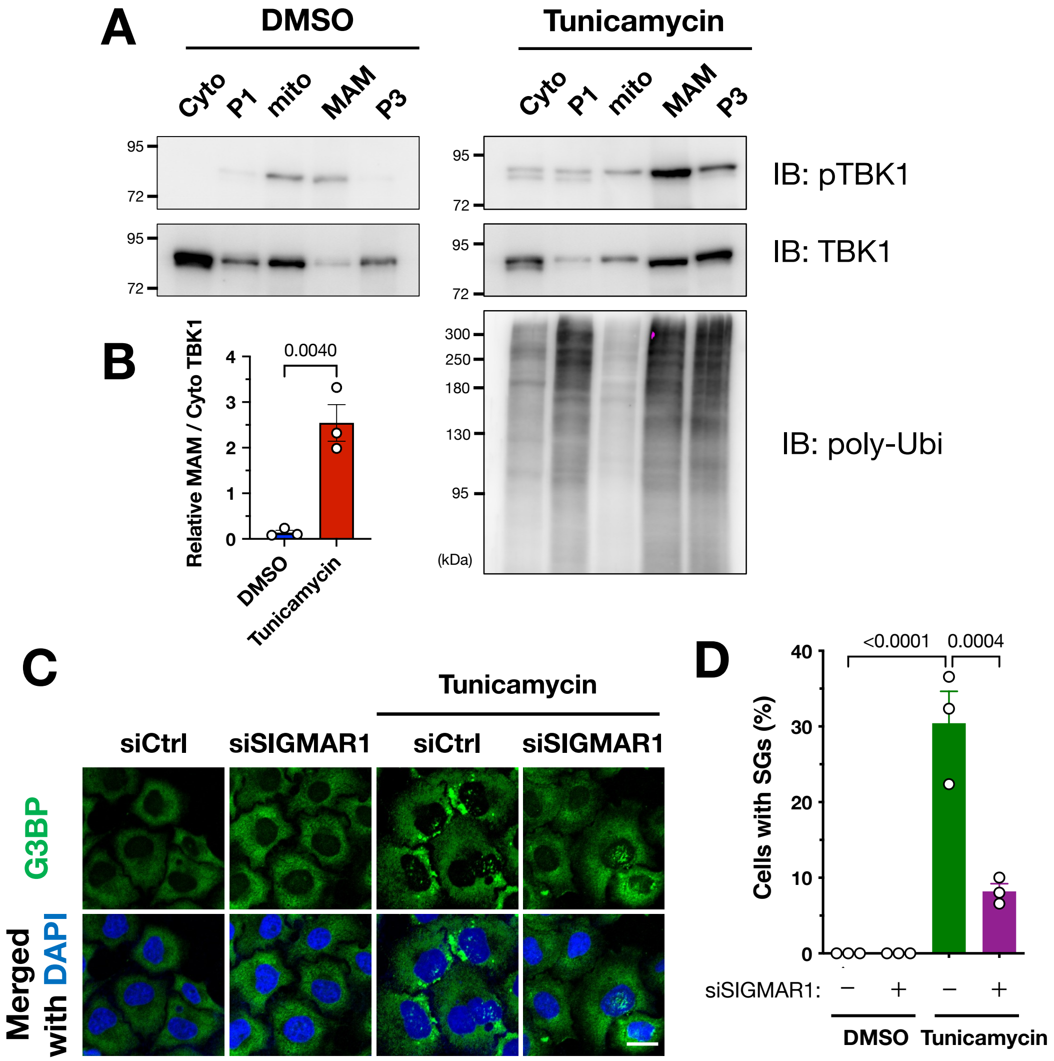

Fig. S14. Tunicamycin, another proteostatic stressor, recapitulated the MAM-dependent cellular response to arsenite treatment. HeLa cells were treated with 10 µg/mL tunicamycin for 6 hours. Representative immunoblotting images of fractionated HeLa cells and quantified relative TBK1 levels at the MAM normalized to cytoplasmic TBK1 levels are shown in (A) and (B), respectively. SGs in siCtrl or siSIGMAR1-treated Hela cells were visualized by immunocytochemistry using anti-G3BP antibody (C) and quantified the percentage of the cells with SGs. The data are expressed as mean ± SEM, analyzed by Student's t-tests (B) or one-way ANOVA following Tukey's multiple comparison tests (D), and p-values are shown as the numbers. Scale bar: 10 µm.

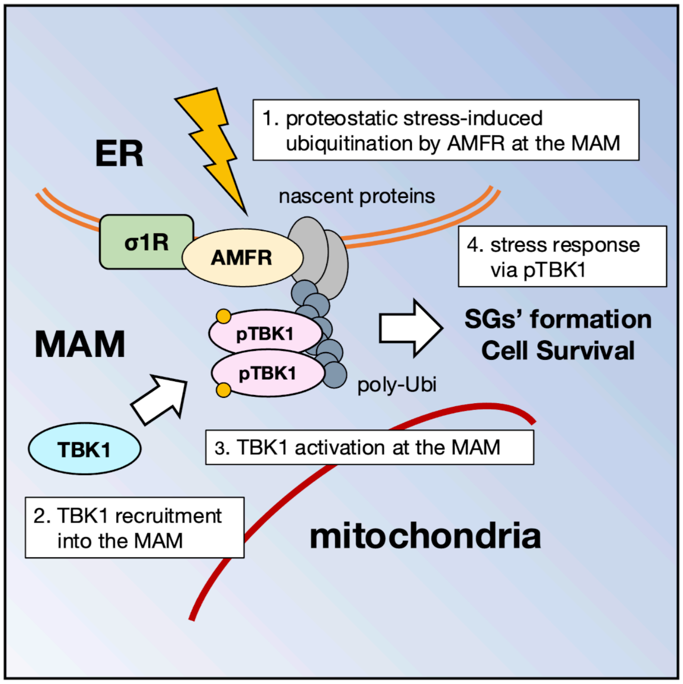

Fig. S15. Summary of cellular response against proteostatic stress via the MAM–TBK1 axis.

Table S1. Primary antibodies used in this study

Product Name	RRID	Cat#	Manufacturer	Dilution for immuno-blotting	Dilution for immuno-staining
Anti-phosphoTBK1/NAK (Ser172) (D52C2)	AB_10693472	5483	Cell Signaling Technology (CST)	1:1000	1:100
Anti-TBK1-NAK	AB_2199749	3013	CST	1:1000	Not used
Anti-β-actin	AB_591279	5441	Sigma-Aldrich	1:5000	Not used
Anti-Choline acetyltransferase (ChAT)	AB_2079751	AB144P	EMD Millipore	Not used	1:100
Anti-sigma receptor (L-20) *discontinued	AB_670292	sc-16203	Santa Cruz	1:250	1:50
Anti-SIIGMAR1 (D4J2E)	AB_2799617	61994	CST	1:1000	1:250
Anti-NeuN	AB_2298767	MAB377	EMD Millipore	Not used	1:100
Anti-fibrillarin (C13C3)	AB_2778067	61994	CST	1:1000	Not used
Anti-Tomm20 (D8T4N)	AB_2687663	42406	CST	1:1000	Not used
Anti-human G3BP [23G3BP(RUO)]	AB_398437	611126	BD biosciences	Not used	1:100
Anti-GAPDH (6C5)	AB_2107445	MAB374	EMD Millipore	1:500	Not used
Anti-Myc tag	AB_591116	562	Medical & Biological Laboratories (MBL)	1:1000	Not used
Anti-multi-Ubiquitin	AB_592937	D058-3	MBL	1:1000	Not used
Anti-V5 tag	AB_2556554	R960-25	Thermo Fisher	1:1000	Not used
Anti-ribosomal protein S3 (RPS3) (D50G7)	AB_10622038	9538	CST	1:1000	Not used
Anti-ribosomal protein L26 (RPL26)	AB_2146242	2065	CST	1:1000	Not used
Anti-glial fibrillary acidic protein (GFAP) (G-A-5)	AB_477010	G3893	Sigma-Aldrich	Not used	1:400
Anti-Iba1	AB_839504	019-19741	FUJIFILM Wako pure chemistry (Wako)	Not used	1:1000

Table S2. Summary of clinical information of the patients with ALS or other neurological diseases

Case	Clinical diagnosis	Age	sex	Disease duration	Post mortem interval
Control-1	MSA-c	75	M	-	N/A
Control-2	Stroke	74	M	-	16h
Control-3	FAP	71	M	-	1h
Control-4	PD	78	F	-	2h
ALS-1	ALS	71	M	2 years 9 months	12h
ALS-2	ALS/FTLD	66	M	3 years	13h
ALS-3	ALS	75	F	2 years 10 months	3h
ALS-4	ALS/FTLD	68	M	1 year 11 months	N/A

N/A, not available; M, male; F, female; ALS, amyotrophic lateral sclerosis
MSA-c, Multiple system atrophy, cerebellar type; FAP, familial amyloidotic polyneuropathy;
PD, Parkinson's disease; FTLD, frontotemporal lobar degeneration.

SI References

1. T. Taniguchi et al., Failure of germinal center formation and impairment of response to endotoxin in tumor necrosis factor alpha-deficient mice. Lab Invest. 77, 647-658 (1997).
2. S. Watanabe et al., Mitochondria-associated membrane collapse is a common pathomechanism in SIGMAR1- and SOD1-linked ALS. *EMBO Mol. Med.* 8, 1421-1437 (2016).
3. Y. S. Tan, Y. L. Lei, Generation and Culture of Mouse Embryonic Fibroblasts. Methods Mol. Biol. 1960, 85-91 (2019).
4. K. Motohashi, Seamless Ligation Cloning Extract (SLiCE) Method Using Cell Lysates from Laboratory Escherichia coli Strains and its Application to SLiP Site-Directed Mutagenesis. *Methods Mol. Biol.* 1498, 349-357 (2017).
5. S. Y. Lee et al., APEX Fingerprinting Reveals the Subcellular Localization of Proteins of Interest. *Cell Rep.* 15, 1837-1847 (2016).
6. T. Hayashi, A. Lewis, E. Hayashi, M. J. Betenbaugh, T. P. Su, Antigen retrieval to improve the immunocytochemistry detection of sigma-1 receptors and ER chaperones. *Histochem. Cell Biol.* 135, 627-637 (2011).
7. V. M. Rodríguez, L. Carrizales, M. E. Jiménez-Capdeville, L. Dufour, M. Giordano, The effects of sodium arsenite exposure on behavioral parameters in the rat. *Brain Res. Bull.* 55, 301-308 (2001).
8. K. Nishino et al., Mice deficient in the C-terminal domain of TAR DNA-binding protein 43 develop age-dependent motor dysfunction associated with impaired Notch1-Akt signaling pathway. *Acta Neuropathol. Commun.* 7, 118 (2019).
